# Prevalence, risk factors, and management practices of premenstrual syndrome among female university students in Lebanon: An observational cross-sectional study

**DOI:** 10.1371/journal.pone.0354807

**Published:** 2026-07-27

**Authors:** Rania Itani, Reem Al Rawass, Diala Tlayss, Fatima Al Zarif, Souraya Domiati, Chaza Alaeddine, Mohamad Abdallah, Rawad Hayouk

**Affiliations:** 1 Pharmacy Practice Department, Faculty of Pharmacy, Beirut Arab University, Beirut, Lebanon; 2 Department of Pharmacology and Therapeutics, Faculty of Pharmacy, Beirut Arab University, Beirut, Lebanon; Dana-Farber Cancer Institute, Harvard Medical School and THE Broad Institute of MIT and Harvard, LEBANON

## Abstract

**Background:**

Premenstrual syndrome (PMS) is common among women of reproductive age and may impair quality of life, academic performance, and social functioning. In Lebanon, data on PMS and its management among female university students remain limited. This study aimed to estimate the prevalence of PMS among female university students in Lebanon, identify risk factors, and recognize adopted management practices and their perceived effectiveness.

**Methods:**

An observational cross-sectional study was conducted among female students at Beirut Arab University, Lebanon, between April 20 and May 8, 2026. Data were collected using a self-administered questionnaire assessing sociodemographic, menstrual, lifestyle, behavioral, psychosocial, and management-related factors. Premenstrual symptoms, social media addiction, and perceived stress were assessed using the Premenstrual Symptoms Screening Tool, Bergen Social Media Addiction Scale, and Perceived Stress Scale-4, respectively. Binary logistic regression was used to identify factors associated with moderate-to-severe PMS and premenstrual dysphoric disorder (PMDD).

**Results:**

Among 1,062 participants, 497 participants screened positive for moderate-to-severe PMS (46.8%), and 175 had symptoms consistent with PMDD based on the PSST (16.5%). The most frequently reported moderate-to-severe symptoms were physical symptoms (69.7%), fatigue or lack of energy (68.8%), depressed mood or hopelessness (67.8%), and overeating or food cravings (66.2%). Academic absenteeism was reported by 37.2%. More than one-third of the participants with moderate-to-severe PMS and PMDD (39%) reported using pharmacological management practices, mainly non-steroidal anti-inflammatory drugs. However, less than half of medication users perceived these treatments as being very effective (45.8%). Higher odds of moderate-to-severe PMS were observed among current smokers (AOR = 1.61, P = 0.003), those with heavy menstrual bleeding (AOR = 1.58, P = 0.03), high meal skipping (AOR = 1.38, P = 0.03), high fast-food consumption (AOR = 1.34, P = 0.04), higher social media addiction scores (AOR = 1.31, P < 0.001), and higher perceived stress scores (AOR = 1.48, P < 0.001).

**Conclusion:**

PMS was common among female university students in Lebanon and a considerable proportion experienced PMDD. In fact, lifestyle, behavioral, and psychosocial factors were associated with moderate-to-severe symptoms, highlighting the need for university-based awareness, screening, and counseling strategies that address modifiable risk factors and support appropriate management of premenstrual symptoms.

## Introduction

Premenstrual syndrome (PMS) is a common gynecological condition affecting women of reproductive age [1,2]. According to the American College of Obstetricians and Gynecologists (ACOG), PMS is characterized by a combination of physical, emotional, and behavioral symptoms that occur during the five days prior to menstruation and resolve within four days after the onset of menstrual flow, persisting for at least three consecutive menstrual cycles [3]. These symptoms vary widely and may include mood changes, anxiety, irritability or anger, sleep disturbances, fatigue, and physical complaints such as abdominal cramps and bloating [1,3].

PMS is associated with a significant decline in quality of life, affecting both functional and psychosocial well-being [4,5]. Women experiencing PMS often report increased absenteeism from work or university as well as reduced academic or occupational performance, largely due to difficulties in concentration, decreased productivity, and fatigue [5,6]. PMS severity is determined by symptom intensity and functional impairment [1,4,7]. PMDD represents the severe end of the premenstrual symptom spectrum and is associated with substantial impairment in quality of life [1,4,5].

Globally, PMS affects 20–30% of reproductive-aged women [8]. However, reported prevalence differs markedly by region: approximately 85% in Africa, 60% in South America, 46% in Asia, and 40% in Europe [9]. Country-specific data further illustrate this variability, ranging from 92.3% in Jordan [10], 80.2% in Egypt [11], 71.9% in Palestine [12], and 66.3% in Syria [13]. In Lebanon, the prevalence of PMS has been reported to be approximately 63% among university students, indicating a high local burden [14].

The etiology of PMS is multifactorial and involves neurochemical and hormonal mechanisms [15,16]. Evidence suggests that symptoms may reflect increased sensitivity to cyclical hormonal changes rather than abnormal progesterone levels [2,16]. Fluctuations in estrogen, progesterone, serotonin, and the progesterone metabolite allopregnanolone may contribute to affective symptoms through altered GABAergic transmission during the luteal phase [15,16].

Several sociodemographic factors, including age, marital status, educational level, and body mass index (BMI), have been identified as risk factors for PMS [17]. In addition, menstrual pattern characteristics such as age at menarche, cycle length and flow, and duration of menstruation are strongly implicated in PMS, reflecting underlying hormonal fluctuations that contribute to symptom development [1,18,19]. Meal skipping, which is common among teenagers, and high consumption of fast food have been linked to PMS [19,20]. Apart from that, the increasing use of social media, especially among university students, raises concern since excessive social media consumption is associated with PMS symptoms [3]. Other modifiable factors, including physical inactivity, smoking, stress, and sleep disturbances, are also associated to a higher risk and greater severity of PMS [18,21].

Management of PMS includes lifestyle modifications, pharmacological treatment, and complementary therapies, with the aims of reducing symptoms, improving functioning, and enhancing quality of life [22,23]. Non-pharmacological approaches, including smoking cessation, regular exercise, dietary modifications, stress management, behavioral therapy, and selected complementary therapies, are commonly recommended for mild-to-moderate symptoms; however, evidence for herbal remedies and supplements remains variable [23–26]. Pharmacological treatment is generally reserved for persistent or more severe symptoms and may include NSAIDs, SSRIs, hormonal contraceptives, anxiolytics, and, in selected cases, SNRIs [7,10]. SSRIs are considered particularly effective for mood-related symptoms and may be used continuously or during the luteal phase, while combined oral contraceptives containing drospirenone and ethinyl estradiol may help alleviate physical symptoms by suppressing ovulation and reducing hormonal fluctuations [7,10–13].

Studies from Saudi Arabia and Turkey suggest that many women with PMS rely on self-management rather than professional consultation. Pharmacological treatment, particularly NSAIDs and other pain relievers, is commonly used, whereas non-pharmacological approaches such as rest and warm compresses also remain frequent [1,27,28].

Despite its burden, PMS remains under-recognized in Lebanon, and local evidence regarding management-seeking behaviors and the perceived effectiveness of management strategies among female university students is limited. Therefore, this study aimed to estimate the prevalence of moderate-to-severe PMS and symptoms consistent with PMDD among female university students in Lebanon, identify associated factors, and describe management practices and their perceived effectiveness.

## Methods

### Study design and setting

An observational cross-sectional study using convenience sampling was conducted among female students at Beirut Arab University, Lebanon, from April 20 to May 8, 2026. Beirut Arab University has four campuses distributed across Lebanon, and data were collected from students at all four campuses. The university enrolls students from diverse Lebanese regions and socioeconomic and cultural backgrounds.

### Inclusion and exclusion criteria

The inclusion criteria were female students enrolled at Beirut Arab University, aged 18–45 years. Participants were excluded if they reported gynecological conditions such as endometriosis, uterine fibroids, uterine polyps, polycystic ovary syndrome (PCOS), chronic pelvic inflammatory disease, or amenorrhea. Additionally, females reporting medical conditions, including thyroid disorders or diagnosed mental health conditions (e.g., anxiety or depression), as well as those currently using hormonal medications for contraception, were excluded. Pregnant or breastfeeding women were also excluded.

### Questionnaire development and structure

A self-administered questionnaire was developed by the research team following a review of relevant literature [4,5,7,29]. It consisted of 39 closed-ended questions covering sociodemographic characteristics, menstrual history, premenstrual symptoms, lifestyle and psychosocial factors, and PMS management practices. The questionnaire was administered in English, and the PSST, BSMAS, and PSS-4 were used in their original English versions. The full questionnaire is provided in S1 Appendix.

Premenstrual symptoms were assessed using the Premenstrual Symptoms Screening Tool (PSST), a validated instrument for screening PMS and PMDD [29]. The PSST includes 14 symptom items and 5 functional-impairment items, each rated on a 4-point Likert scale from 0 (“not at all”) to 3 (“severe”). PMS and PMDD were classified according to the established PSST criteria based on symptom number, severity, and functional impairment [29].

Social media addiction severity was assessed using the 6-item Bergen Social Media Addiction Scale (BSMAS), with total scores ranging from 6 to 30 and higher scores indicating greater risk of addictive social media use [30,31]. Perceived stress was measured using the 4-item Perceived Stress Scale (PSS-4), with total scores ranging from 0 to 16 and higher scores indicating greater perceived stress [32,33]. The final section assessed healthcare consultation, non-pharmacological strategies, medication use, perceived effectiveness, and safety.

Five experts in clinical pharmacy, pharmacy practice, and pharmacology reviewed the questionnaire for face and content validity. A pilot test was conducted among 20 university students to assess clarity, organization, completion time, and reproducibility; pilot data were not included in the final analysis.

### Sample size calculation

The sample size was calculated using the standard formula for cross-sectional studies developed by Daniel et al., where n was the required sample size, Z was the Z-value corresponding to a 95% confidence level (1.96 at α = 0.05), P was the estimated prevalence of PMS (63%) [14], and d was the desired precision (4%) [34]. Based on this calculation, the minimum required sample size was 358.


$$\begin{array}{c} n=\frac{Z^{\text{2}}\;\;P(\text{1}-P)}{d^{\text{2}}} \end{array}$$

### Data collection and data analysis

Three trained researchers recruited eligible female students from university common areas. After receiving information about the study and providing electronic informed consent, participants completed an anonymous self-administered questionnaire on a tablet device. Participation was voluntary, and participants could withdraw before submission. The questionnaire required approximately 10 minutes to complete.

All submitted questionnaires contained complete responses to mandatory items. Data were screened for consistency and eligibility before coding and analysis. Data were coded and analyzed using IBM SPSS Statistics version 24. Categorical variables are presented as frequencies and percentages, and continuous variables as means ± standard deviations. Analyses of PMS management practices and perceived effectiveness were restricted to participants with moderate-to-severe PMS or PMDD.

Associations with moderate-to-severe PMS/PMDD were initially assessed using Pearson’s chi-square test. Variables with P < 0.20 in univariate analyses and clinically relevant variables were entered into a multivariable binary logistic regression model using the Enter method. Model fit was assessed using the Hosmer–Lemeshow test. Adjusted odds ratios with 95% confidence intervals were reported, and P < 0.05 was considered statistically significant.

### Operational definitions

**Academic specialization:** Participants enrolled in various faculties and majors were classified as either medical or non-medical students. Medical students (from the faculties of pharmacy, medicine, nursing, dentistry and applied health sciences) were expected to possess medical knowledge regarding common health conditions. In contrast, non-medical students included those studying in faculties outside the medical field.

### Ethical consideration

The study was designed and conducted in accordance with the ethical principles outlined in the World Medical Association Declaration of Helsinki [35]. Ethical approval was obtained from the Institutional Review Board (IRB) at Beirut Arab University on April 17, 2026. No approval code was issued at the time of approval. The IRB confirmed that the study was reviewed and approved in accordance with institutional regulations and guidelines, and that data collection was authorized to proceed.

Participants were provided with a clear explanation of the purpose and procedures of the study prior to participation. Participants who agreed to take part provided electronic informed consent via a tablet-based questionnaire before completing the survey. Participation was entirely voluntary, and participants were informed that they could refuse or withdraw at any time prior to submission of their responses without any consequences. Confidentiality, anonymity, and non-traceability were strictly maintained throughout the study.

## Results

### Participant enrollment and study population

**Fig 1:**
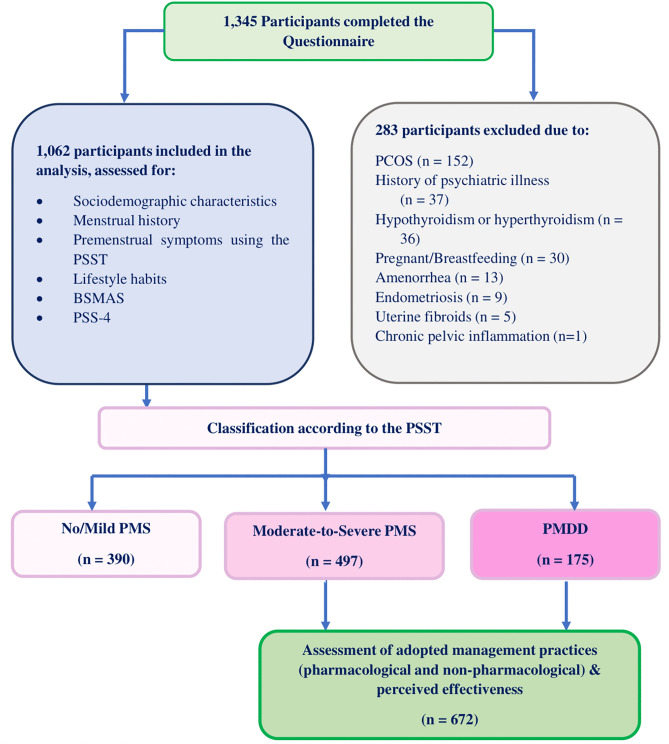
Flowchart of Participant Enrollment, Classification, and Study Analysis. BSMAS: Bergen Social Media Addiction Scale; PCOS: Polycystic Ovary Syndrome; PMS: Premenstrual Syndrome; PSST: Premenstrual Symptoms Screening Tool; PMDD: Premenstrual Dysphoric Disorder; PSS-4: Perceived Stress Scale-4.

A total of 1,345 female participants completed the electronic questionnaire. Of these, 283 participants were excluded for not meeting the inclusion criteria, including participants with PCOS (n = 152), history of psychiatric illness (n = 37), hypothyroidism or hyperthyroidism (n = 36), pregnancy or breastfeeding (n = 30), amenorrhea (n = 13), endometriosis (n = 9), uterine fibroids (n = 5), and chronic pelvic inflammation (n = 1). Therefore, 1,062 participants were included in the final analysis. The electronic questionnaire required completion of all mandatory items before submission; thus, no questionnaires were excluded because of incomplete responses or missing data. Further details regarding participant enrollment and analysis are presented in Fig 1.

### Sociodemographic characteristics of the participants

The mean age of the participants was 22.09 ± 4.58 years, with ages ranging from 18 to 45 years. Nearly half of the participants were aged between 18 and 20 years (n = 499, 47.0%), followed by those aged 21–24 years (n = 361, 34%). Concerning the academic specialization, more than half of the participants were enrolled in non-medical faculties (n = 565, 53.2%). As for the academic level, almost half of the participants were registered in the second (n = 238, 22.4%) and third academic years (n = 243, 22.9%), with a notable portion also pursuing a Master’s degree (n = 158, 14.9%). The majority of participants were single or engaged (n = 969, 91.2%). The mean BMI was 23.92 ± 4.21 kg/m^2^, with more than half of the participants having normal weight (56%), while 29.2% were overweight and 7.7% were obese (Table 1).

**Table 1:** Participants’ sociodemographic data (N = 1062).

Item	n (%)
	
**Age (years)**	
Mean ± standard deviation: 22.09 ± 4.58 years (Range:18–45)	
18–20	499 (47)
21–24	361 (34)
25–30	140 (13.2)
> 30	62 (5.8)
**Academic level**	
Freshman	40 (3.8)
First	201 (18.9)
Second	238 (22.4)
Third	243 (22.9)
Fourth	77 (7.3)
Fifth	55 (5.2)
Sixth	11 (1)
PharmD	24 (2.3)
Master’s/MBA	158 (14.9)
PhD/DBA	15 (1.4)
**Academic Specialization**	
Medical	497 (46.8)
Non-medical	565 (53.2)
**Marital status**	
Single/engaged	969 (91.2)
Divorced	8 (0.8)
Married	83 (7.8)
Widowed	2 (0.2)
**Weight (kg)**	
Mean ± standard deviation: 63.06 ± 11.26 kg (Range: 40–120)	
**Height (cm)**	
Mean ± standard deviation: 162.54 ± 5.61 cm (Range: 145–181)	
**BMI (Kg/m**^**2**^)	
Mean ± standard deviation: 23.92 ± 4.21 kg/m^2^ (Range: 15.62–37.18)	

**Note:** Academic level: “Freshman” refers to students enrolled in the preparatory foundation year before entry into an undergraduate degree program, whereas “First year” refers to the first year of an undergraduate degree program.

### Menstrual characteristics of the participants

The mean age at menarche was 12.35 ± 1.29 years, with most participants reporting normal menarche between 12 and 13 years (n = 628, 59.1%). The mean menstrual cycle duration was 28.26 ± 3.57 days, and the majority had a normal cycle duration of 24–38 days (n = 956, 90%). Regarding menstrual flow, the mean duration was 6.16 ± 1.29 days, with most participants reporting a normal flow duration of 4.5–8 days (n = 944, 88.9%). In terms of menstrual bleeding intensity, moderate bleeding (more than 1 pad soaked in 3 hours) was the most reported pattern (n = 748, 70.4%) (Table 2).

**Table 2:** Participants’ menstrual characteristics (N = 1,062).

Item	n (%)
**Age at Menarche (years)**	
Mean ± standard deviation: 12.35 ± 1.29 (Range: 9–16)	
< 12 years (early menarche)	246 (23.2)
12–13 years (normal menarche)	628 (59.1)
> 13 years (late menarche)	188 (17.7)
**Menstrual Cycle duration (days)**	
Mean ± standard deviation: 28.26 ± 3.57 (Range: 19–40)	
< 24 days (frequent)	89 (8.4)
24–38 days (normal)	956 (90)
> 38 days (infrequent)	17 (1.6)
**Menstrual Flow duration (days)**	
Mean ± standard deviation: 6.16 ± 1.29 (Range: 1–10)	
< 4.5 d (shortened)	91 (8.6)
4.5–8 d (normal)	944 (88.9)
> 8 d (prolonged)	27 (2.5)
**Menstrual Bleeding Intensity**	
Light (<1 pad soaked in 3 hours)	141 (13.3)
Moderate (>1 pad soaked in 3 hours)	748 (70.4)
Heavy (>1 pad soaked every 2 hours)	173 (16.3)

When participants were asked whether they believed they might have PMS, the majority acknowledged this fact (n = 946, 89.1%).

### Premenstrual symptoms among participants

S1 Table demonstrates the frequency and severity of premenstrual symptoms experienced by participants assessed using the PSST. Physical symptoms, including breast pain, headache, muscle pain, swollen stomach, and weight gain, had the highest mean score (1.94 ± 0.92) out of 4, with nearly two-thirds of participants reporting moderate-to-severe symptoms (n = 740, 69.7%). Fatigue or lack of energy, and depressed mood/hopelessness were also highly reported symptoms, with 68.8% and 67.8% of the participants reporting moderate-to-severe symptoms, respectively. Additionally, overeating or food craving were commonly reported, with 66.2% of participants experiencing moderate-to-severe symptoms. In contrast, insomnia had the lowest mean score (0.8 ± 0.92), with nearly half of the participants reporting no insomnia symptoms (n = 509, 47.9%).

### Interference of premenstrual symptoms with daily life

S2 Table presents the extent to which premenstrual symptoms interfered with participants’ daily life activities. Home responsibilities showed the greatest level of interference, with a mean score of 1.48 ± 0.97 out of 4, where 50.5% reported moderate-to-severe interference. Social life activities and study efficiency or productivity were also commonly affected, where 44.6% and 43.2% of the participants reported moderate-to-severe interference, respectively. Relationships with colleagues demonstrated the lowest interference score (1 ± 0.86), with one-third of participants reporting no interference at all (n = 356, 33.5%).

### Severity classification of premenstrual symptoms

**Fig 2:**
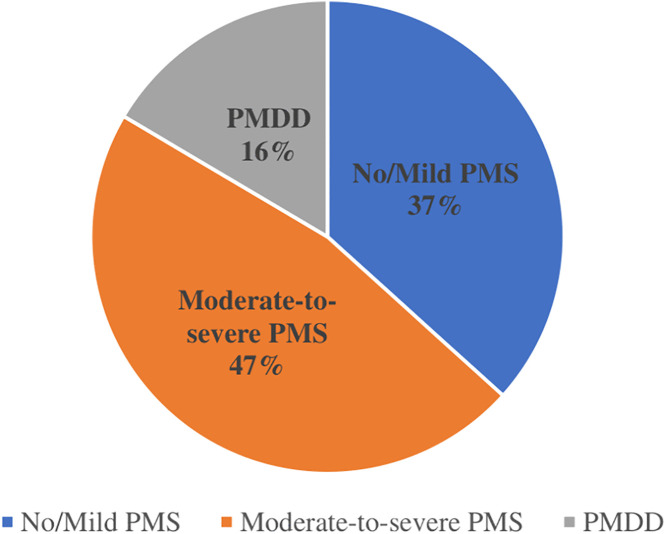
Severity classification of premenstrual symptoms.

According to the PSST classification criteria, nearly half of the participants screened positive for moderate-to-severe PMS based on the PSST (n = 497, 46.8%), while 390 participants (36.7%) were classified as having no/mild PMS. Notably, 175 participants (16.5%) met the criteria for PMDD, as illustrated in Fig 2.

### Academic absenteeism due to PMS symptoms

When participants were asked about the academic absenteeism related to PMS symptoms, more than one-third reported missing academic activities due to their premenstrual symptoms (n = 395, 37.2%).

### Lifestyle patterns among participants

As shown in **Table 3**, out of 1,062 participants, one-third were current smokers (n = 340, 32%), while 35 were ex-smokers (3.3%). Concerning the type of smoking, more than half of the smokers reported smoking hookah (n = 209, 61.5%), whereas 27 participants (7.9%) reported smoking cigarettes. In terms of cigarette consumption, 25.9% smoked 6–10 cigarettes per day, while 37% smoked more than 11 cigarettes per day. Among the hookah smokers, 18.7% of the participants reported smoking 3 hookah sessions, whereas 6.7% reported smoking 4 sessions per day. More than half of the female participants reported either not engaging in physical exercise or exercising only occasionally (n = 610,57.5%). Moreover, the mean sleep duration was 7.57 ± 1.68 hours, ranging from 3 to 15 hours. Short sleep duration (<7 hours) was noted by 292 participants (27.5%), while 153 participants (14.4%) reported long sleep duration (>9 hours). Furthermore, 430 participants (40.5%) experienced sometimes skipping meals, whereas 202 participants (19%) reported frequent meal skipping. In addition, nearly half of the participants reported sometimes consuming fast food (n = 472, 44.4%), whereas 178 participants (16.8%) reported frequent fast-food consumption.

**Table 3:** Lifestyle habits among participants (N = 1,062).

Item	n (%)
**Smoking Status**	
Never Smoker	687 (64.7)
Current Smoker	340 (32)
Ex-smoker	35 (3.3)
**Smoking Methods** ^**a**^	
Cigarette	27 (7.9)
Hookah (nargila)	209 (61.5)
Electronic cigarettes/vape/IQOS	104 (30.6)
**Cigarette Consumption /Day**	
5 or less	10 (37)
6 −10	7 (25.9)
11-20	4 (14.8)
21-30	5 (18.5)
31 or more	1 (3.7)
**Hookah Sessions/Day**	
1	99 (47.4)
2	54 (25.8)
3	39 (18.7)
4	14 (6.7)
5	1 (0.5)
6 or more	2 (1)
**Physical Exercise**	
I do not exercise	279 (26.3)
Occasionally	331 (31.2)
1–2 times per week	200 (18.8)
3–4 times per week	167 (15.7)
5–6 times per week	46 (4.3)
Daily	39 (3.7)
**Sleep Duration**	
Mean ± standard deviation: 7.57 ± 1.68 (Range: 3–15 h)	
Short sleep (<7 h)	292 (27.5)
Normal sleep (7–9 h)	617 (58.1)
Long sleep (>9 h)	153 (14.4)
**Meal Skipping**	
Rarely/ Never	228 (21.5)
Sometimes	430 (40.5)
Occasionally	202 (19)
Frequently	202 (19)
**Fast Food Consumption**	
Rarely/ Never	143 (13.5)
Sometimes	472 (44.4)
Occasionally	269 (25.3)
Frequently	178 (16.8)

^a^Out of the total number of current smokers 340

**Fig 3:**
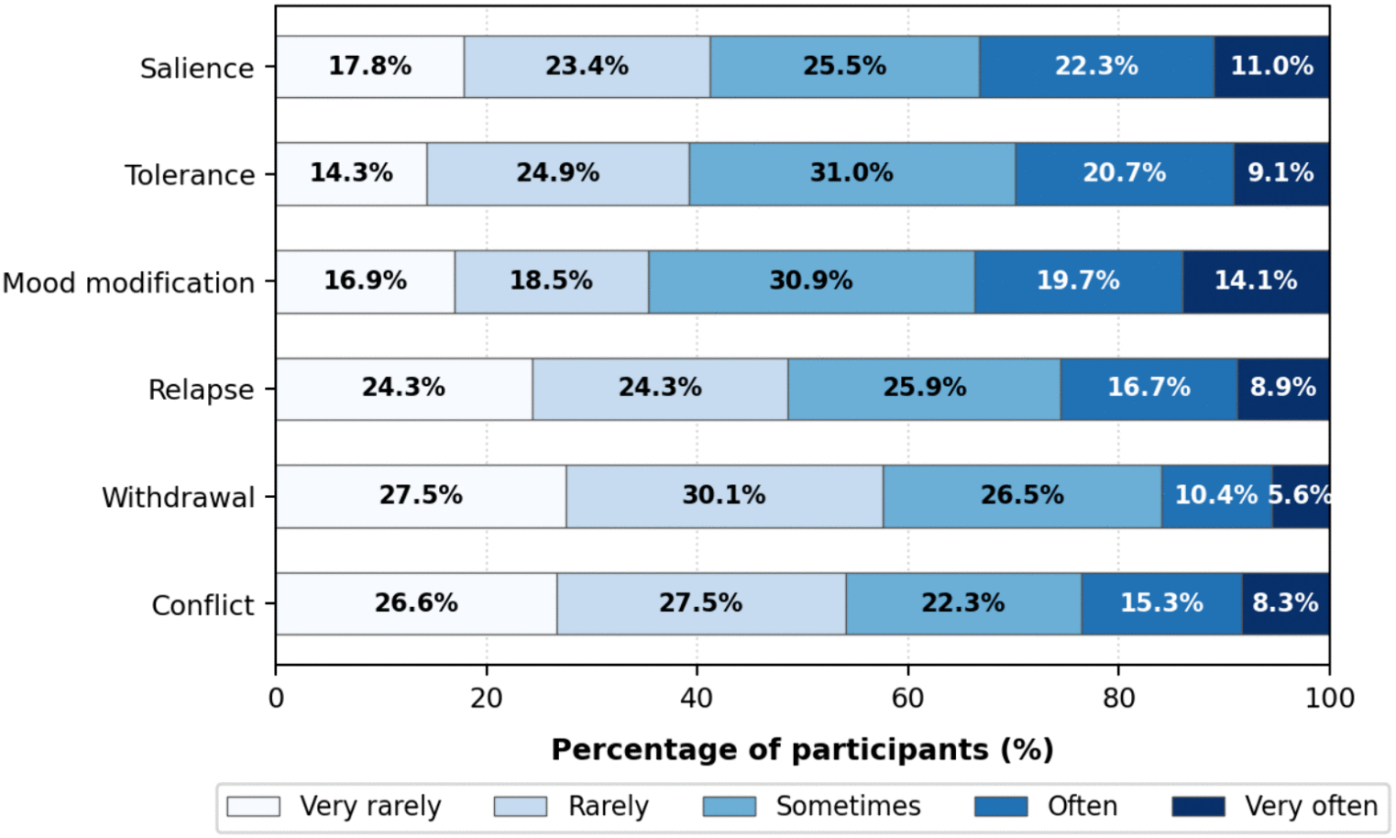
Distribution of participants’ responses to the Bergen Social Media Addiction Scale (BSMAS) items. Responses are presented as percentages for each response category (Very rarely, Rarely, Sometimes, Often, and Very often). Higher frequencies of “Often” and “Very often” indicate greater endorsement of social media addiction-related behaviors. BSMAS = Bergen Social Media Addiction Scale.

**Fig 4:**
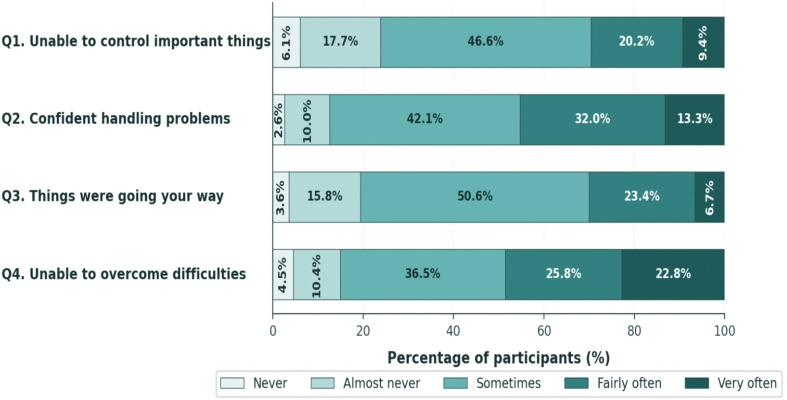
Distribution of participants’ responses to the Perceived Stress Scale-4 (PSS-4) items. Responses are presented as percentages of participants for each PSS-4 item.

The mean BSMAS score was 16.16 ± 5.71, while the mean PSS-4 score was 8.04 ± 2.10. Fig 3 and Fig 4 present the distribution of participants’ responses to the individual items of the BSMAS and PSS-4, respectively.

### Predictors associated with moderate-to-severe PMS/PMDD

Table 4 presents the univariate analysis of potential predictors associated with moderate-to-severe PMS/PMDD among female university students. Compared with students aged 18–20 years, those aged 25–30 years (UOR = 0.49, 95% CI = 0.34–0.72, P < 0.001) and those older than 30 years (UOR = 0.46, 95% CI = 0.27–0.79, P < 0.001) had significantly lower odds of moderate-to-severe PMS/PMDD. Academic level was also significantly associated with the outcome, with advanced undergraduate/professional students (UOR = 0.75, 95% CI = 0.57–0.98, P = 0.04) and postgraduate students (UOR = 0.63, 95% CI = 0.44–0.89, P = 0.01) showing lower odds compared with early undergraduate students. Ever- students were also less likely to have moderate-to-severe PMS/PMDD compared with single/engaged students (UOR = 0.56, 95% CI = 0.37–0.86, P = 0.01).

**Table 4:** Univariate analysis of potential predictors associated with moderate-to-severe PMS/PMDD (N = 1,062).

Item	n (%)^a^	PMS/PMDD status		UOR (95% CI)	*P* ^*c*^
		No/Mild PMS n = 390 (%)^b^	Moderate-to-severe PMS/PMDD n = 672 (%)^b^		
**Age** (reference: 18–20 years)^**†**^					
18-20 years	499 (47)	165 (33.1)	334 (66.9)	–	–
21-24 years	361 (34)	123 (34.1)	238 (65.9)	0.96 (0.72-1.27)	0.76
25-30 years	140 (13.2)	70 (50)	70 (50)	0.49 (0.34-0.72)	<0.001^d^
>30 years	62 (5.8)	32 (51.6)	30 (48.4)	0.46 (0.27-0.79)	<0.001^d^
**Academic specialization** (reference: non-medical)					
Non-medical	565 (53.2)	214 (37.9)	351 (62.1)	–	0.41
Medical/health-related	497 (46.8)	176 (35.4)	321 (64.6)	1.11 (0.87-1.43)	
**Academic level** (reference: early undergraduate)^**†**^					
Early undergraduate	479 (45.1)	155 (32.4)	324 (67.6)	–	–
Advanced undergraduate/professional	410 (38.6)	160 (39.0)	250 (61.0)	0.75 (0.57-0.98)	0.04^d^
Postgraduate	173 (16.3)	75 (43.4)	98 (56.6)	0.63 (0.44-0.89)	0.01^d^
**Marital status** (reference: single/engaged)^**†**^					
Single/engaged	969 (91.2)	344 (35.5)	625 (64.5)	–	0.01^d^
Ever married	93 (8.8)	46 (49.5)	47 (50.5)	0.56 (0.37-0.86)	
**Menarche age (years)** (reference: Normal menarche (12–13 years))^**†**^					
Normal menarche (12–13 years)	628 (59.1)	223 (35.5)	405 (64.5)	–	–
Early menarche (<12 years)	246 (23.2)	90 (36.6)	156 (63.4)	0.95 (0.70-1.30)	0.77
Late menarche (>13 years)	188 (17.7)	77 (41.0)	111 (59.0)	0.79 (0.57-1.11)	0.17
**Menstrual cycle duration (days)** (reference: normal cycle (24–38 days))^**†**^					
Normal cycle (24–38 days)	956 (90)	345 (36.1)	611 (63.9)	–	–
Frequent cycle (<24 days)	89 (8.4)	39 (43.8)	50 (56.2)	0.72 (0.47-1.12)	0.15
Infrequent cycle (>38 days)	17 (1.6)	6 (35.3)	11 (64.7)	1.04 (0.38-2.82)	0.95
**Menstrual flow duration (days)** (reference: Normal duration (4.5–8 days))^**†**^					
Normal duration (4.5–8 days)	944 (88.9)	357 (37.8)	587 (62.2)	–	–
Shortened duration (<4.5 days)	91 (8.6)	27 (29.7)	64 (70.3)	1.44 (0.90-2.30)	0.12
Prolonged duration (>8 days)	27 (2.5)	6 (22.2)	21 (77.8)	2.13 (0.85-5.32)	0.10
**Menstrual bleeding intensity** (reference: Moderate (>1 pad soaked in 3 hours))^**†**^					
Light (<1 pad soaked in 3 hours)	141 (13.3)	65 (46.1)	76 (53.9)	0.70 (0.49-1.01)	0.05
Moderate (>1 pad soaked in 3 hours)	748 (70.4)	280 (37.4)	468 (62.6)	–	–
Heavy (>1 pad soaked every 2 hours)	173 (16.3)	45 (26.0)	128 (74.0)	1.70 (1.17-2.47)	<0.001^d^
**Smoking status** (reference: never smoker)^**†**^					
Never smoker	687 (64.7)	284 (41.3)	403 (58.7)	–	–
Current smoker	340 (32.0)	91 (26.8)	249 (73.2)	1.93 (1.45-2.56)	<0.001^d^
Ex-smoker	35 (3.3)	15 (42.9)	20 (57.1)	0.94 (0.47-1.87)	0.86
**Physical exercise** (reference: no exercise)^**†**^					
No exercise	279 (26.3)	88 (31.5)	191 (68.5)	–	–
Occasional exercise	331 (31.2)	109 (32.9)	222 (67.1)	0.94 (0.67-1.32)	0.71
Regular exercise	452 (42.6)	193 (42.7)	259 (57.3)	0.62 (0.45-0.85)	<0.001^d^
**Sleep duration** (reference: normal sleep (7–9 h))^**†**^					
Normal sleep (7–9 h)	617 (58.1)	244 (39.5)	373 (60.5)	–	–
Short sleep (<7 h)	292 (27.5)	104 (35.6)	188 (64.4)	1.18 (0.89-1.58)	0.26
Long sleep (>9 h)	153 (14.4)	42 (27.5)	111 (72.5)	1.73 (1.17-2.55)	0.01^d^
**Meal skipping** (reference: low meal skipping)^**†**^					
Low meal skipping	658 (62.0)	267 (40.6)	391 (59.4)	–	<0.001^d^
High meal skipping	404 (38.0)	123 (30.4)	281 (69.6)	1.56 (1.20-2.03)	
**Fast food consumption** (reference: Low fast-food consumption)^**†**^					
Low fast-food consumption	615 (57.9)	254 (41.3)	361 (58.7)	–	<0.001^d^
High fast-food consumption	447 (42.1)	136 (30.4)	311 (69.6)	1.61 (1.24-2.08)	
**Bergen Social Media Addiction Scale (BSMAS)** ^**†**^					
BSMAS total score	16.16 ± 5.71	13.79 ± 5.56	17.53 ± 5.34	1.14 (1.11–1.16)	<0.001^d,e^
**PSS-4 total score, mean ± SD** ^**†**^					
PSS-4 total score, mean ± SD	8.04 ± 2.10	7.37 ± 2.11	8.43 ± 1.99	1.30 (1.21–1.39)	<0.001^d^

***CI***, confidence interval; ***UOR***, unadjusted odds ratio.

a Percentages for the column.

b Percentages for the row.

c Univariate binary logistic regression was conducted to assess associations with moderate-to-severe PMS/PMDD.

d Statistically significant (*p* < 0.05).

e Independent t-test was utilized.

† Retained in the final model.

**Note:** Collapsed variables were defined as follows: early undergraduate included freshman, first-year, and second-year students; advanced undergraduate/professional included third-, fourth-, fifth-, sixth-year, and PharmD students; postgraduate included Master’s/MBA and PhD/DBA students. Ever married included married, divorced, and widowed participants. Regular exercise included participants exercising at least 1–2 times per week. Low meal skipping included rarely/never and sometimes, while high meal skipping included occasionally and frequently. Low fast-food consumption included rarely/never and sometimes, while high fast-food consumption included occasionally and frequently. Any war-related impact included displacement from home, financial difficulties due to war, damage or destruction of workplace or home, or loss/injury of a family member.

Regarding menstrual characteristics, heavy menstrual bleeding was significantly associated with higher odds of moderate-to-severe PMS/PMDD compared with moderate bleeding (UOR = 1.70, 95% CI = 1.17–2.47, P < 0.001). Menstrual cycle duration and menstrual flow duration were not significantly associated with the outcome. For lifestyle-related factors, current smokers had significantly higher odds of moderate-to-severe PMS/PMDD compared with never smokers (UOR = 1.93, 95% CI = 1.45–2.56, P < 0.001), while regular exercise was associated with lower odds (UOR = 0.62, 95% CI = 0.45–0.85, P < 0.001). Long sleep duration (> 9 hours/day) was associated with increased odds of moderate-to-severe PMS/PMDD compared with normal sleep duration (UOR = 1.73, 95% CI = 1.17–2.55, P = 0.01). High meal skipping (UOR = 1.56, 95% CI = 1.20–2.03, P < 0.001) and high fast-food consumption (UOR = 1.61, 95% CI = 1.24–2.08, P < 0.001) were also significantly associated with increased odds of moderate-to-severe PMS/PMDD.

Psychosocial factors were strongly associated with moderate-to-severe PMS/PMDD. Higher BSMAS score was significantly associated with increased odds of moderate-to-severe PMS/PMDD (UOR = 1.14, 95% CI = 1.11–1.16, P < 0.001), as was higher PSS-4 total score (UOR = 1.30, 95% CI = 1.21–1.39, P < 0.001).

Multivariable binary logistic regression identified several independent predictors of moderate-to-severe PMS/PMDD (Table 5). Compared with moderate menstrual bleeding, light bleeding was independently associated with lower odds of moderate-to-severe PMS/PMDD (AOR = 0.65, 95% CI = 0.43–0.98, P = 0.04), whereas heavy bleeding was associated with higher odds (AOR = 1.58, 95% CI = 1.05–2.38, P = 0.03). Current smoking remained independently associated with higher odds of moderate-to-severe PMS/PMDD (AOR = 1.61, 95% CI = 1.17–2.21, P = 0.003). High meal skipping (AOR = 1.38, 95% CI = 1.03–1.86, P = 0.03) and high fast-food consumption (AOR = 1.34, 95% CI = 1.00–1.79, P = 0.04) were also independently associated with increased odds of moderate-to-severe PMS/PMDD. In addition, higher BSMAS total score (AOR = 1.31, 95% CI = 1.28–1.34, P < 0.001) and higher PSS-4 total score (AOR = 1.48, 95% CI = 1.38–1.59, P < 0.001) remained significant independent predictors. In contrast, age, academic level, marital status, menarche age, menstrual cycle duration, menstrual flow duration, physical exercise, and sleep duration were not independently associated with moderate-to-severe PMS/PMDD after adjustment.

**Table 5:** Multivariable binary logistic regression^a^ of factors associated with moderate-to-severe PMS/PMDD among female university students.

Predictors	UOR	B	SE	Wald	AOR	95% CI	*p*
**Constant**		−2.87	0.41	50.18	0.057		<0.001^b^
**Age** (reference: 18–20 years)							
21-24 years	0.96	−0.007	0.23	0.001	0.99	0.64-1.55	0.97
25-30 years	0.49	−0.51	0.29	2.94	0.60	0.34-1.08	0.09
>30 years	0.46	−0.73	0.42	3.11	0.48	0.21-1.09	0.08
**Academic level** (reference: early undergraduate)							
Advanced undergraduate/professional	0.75	−0.03	0.22	0.02	0.97	0.63-1.50	0.88
Postgraduate	0.63	0.42	0.30	1.94	1.53	0.84-2.78	0.16
**Marital status** (reference: single/engaged)							
Ever married	0.56	0.02	0.31	0.003	1.02	0.56-1.85	0.95
**Menarche age (years)** (reference: normal menarche (12–13 years))							
Early menarche (<12 years)	0.95	−0.12	0.18	0.50	0.88	0.63-1.24	0.48
Late menarche (>13 years)	0.79	−0.28	0.19	2.11	0.76	0.52-1.10	0.15
**Menstrual cycle duration (days)** (reference: normal cycle (24–38 days))							
Frequent cycle (<24 days)	0.72	−0.46	0.25	3.50	0.63	0.39-1.02	0.06
Infrequent cycle (>38 days)	1.04	−0.50	0.58	0.72	0.61	0.20-1.91	0.40
**Menstrual flow duration (days)** (reference: Normal duration (4.5–8 days))							
Shortened duration (<4.5 days)	1.44	0.45	0.27	2.91	1.57	0.94-2.65	0.09
Prolonged duration (>8 days)	2.13	0.62	0.51	1.43	1.85	0.68-5.07	0.23
**Menstrual bleeding intensity** (reference: moderate bleeding)							
Light (<1 pad soaked in 3 hours)	0.70	−0.44	0.21	4.18	0.65	0.43-0.98	0.04^b^
Heavy (>1 pad soaked every 2 hours)	1.70	0.46	0.21	4.86	1.58	1.05-2.38	0.03^b^
**Smoking status** (reference: never smoker)							
Current smoker	1.93	0.48	0.16	8.57	1.61	1.17-2.21	0.003^b^
Ex-smoker	0.94	0.04	0.39	0.01	1.04	0.49-2.21	0.93
**Physical exercise** (reference: no exercise)							
Occasional exercise	0.94	−0.11	0.19	0.34	0.89	0.61-1.30	0.56
Regular exercise	0.62	−0.31	0.18	3.02	0.73	0.52-1.04	0.08
**Sleep duration** (reference: normal sleep (7–9 h))							
Short sleep (<7 h)	1.18	−0.07	0.17	0.15	0.94	0.67-1.30	0.70
Long sleep (>9 h)	1.73	0.05	0.22	0.05	1.05	0.68-1.62	0.82
**Meal skipping** (reference: low meal skipping)							
High meal skipping	1.56	0.33	0.15	4.60	1.38	1.03-1.86	0.03^b^
**Fast food consumption** (reference: Low fast-food consumption)							
High fast-food consumption	1.61	0.29	0.15	3.92	1.34	1.00-1.79	0.04^b^
**BSMAS total score**							
BSMAS total score	1.14	0.34	0.01	53.86	1.31	1.28-1.34	<0.001ᵇ
**PSS-4 total score**							
PSS-4 total score	1.30	0.39	0.04	34.76	1.48	1.38-1.59	<0.001ᵇ

***AOR***, adjusted odds ratio; ***B***, B, logistic regression coefficient; ***CI***, confidence interval; ***SE***, standard error; ***UOR***, unadjusted odds ratio; ***Wald***, Wald chi-square test that tests the null hypothesis.

a Multivariable binary logistic regression using the Enter method.

b Statistically significant (*p* < 0.05).

### Predictors associated with PMDD

Univariate associations with PMDD are presented in Table 6. In the multivariable analysis, heavy menstrual bleeding, current smoking, regular exercise, higher BSMAS total score, and higher PSS-4 total score were independently associated with PMDD (Table 7).

**Table 6:** Univariate analysis of factors associated with PMDD among female university students (N = 1,062).

Item	n (%)^a^	PMDD		UOR (95% CI)	*P* ^*c*^
**Non-PMDD** n = 887 (%)ᵇ	**PMDD** n = 175 (%)ᵇ
**Age** (reference: 18–20 years)^**†**^					
18-20 years	499 (47)	405 (81.2)	94 (18.8)	–	–
21-24 years	361 (34)	299 (82.8)	62 (17.2)	0.89 (0.63-1.27)	0.53
25-30 years	140 (13.2)	125 (89.3)	15 (10.7)	0.52 (0.29-0.92)	0.02^d^
>30 years	62 (5.8)	58 (93.5)	4 (6.5)	0.30 (0.11-0.84)	0.02^d^
**Academic specialization** (reference: non-medical)^**†**^					
Non-medical	565 (53.2)	480 (85.0)	85 (15.0)	–	0.18
Medical/health-related	497 (46.8)	407 (81.9)	90 (18.1)	1.25 (0.90-1.73)	
**Academic level** (reference: early undergraduate)^**†**^					
Early undergraduate	479 (45.1)	385 (80.4)	94 (19.6)	–	–
Advanced undergraduate/professional	410 (38.6)	347 (84.6)	63 (15.4)	0.74 (0.52-1.06)	0.10
Postgraduate	173 (16.3)	155 (89.6)	18 (10.4)	0.48 (0.28-0.81)	0.01^d^
**Marital status** (reference: single/engaged)					
Single/engaged	969 (91.2)	805 (83.1)	164 (16.9)	–	0.21
Ever married	93 (8.8)	82 (88.2)	11 (11.8)	0.66 (0.34-1.26)	
**Menarche age (years)** (reference: Normal menarche (12–13 years))					
Normal menarche (12–13 years)	628 (59.1)	525 (83.6)	103 (16.4)	–	–
Early menarche (<12 years)	246 (23.2)	208 (84.6)	38 (15.4)	0.93 (0.62-1.40)	0.73
Late menarche (>13 years)	188 (17.7)	154 (81.9)	34 (18.1)	1.13 (0.73-1.73)	0.59
**Menstrual cycle duration (days)** (reference: normal cycle (24–38 days))^**†**^					
Normal cycle (24–38 days)	956 (90)	806 (84.3)	150 (15.7)	–	–
Frequent cycle (<24 days)	89 (8.4)	70 (78.7)	19 (21.3)	1.46 (0.85-2.49)	0.17
Infrequent cycle (>38 days)	17 (1.6)	11 (64.7)	6 (35.3)	2.93 (1.07-8.05)	0.03^d^
**Menstrual flow duration (days)** (reference: Normal duration (4.5–8 days))					
Normal duration (4.5–8 days)	944 (88.9)	794 (84.1)	150 (15.9)	–	–
Shortened duration (<4.5 days)	91 (8.6)	72 (79.1)	19 (20.9)	1.40 (0.82-2.38)	0.22
Prolonged duration (>8 days)	27 (2.5)	21 (77.8)	6 (22.2)	1.51 (0.60-3.81)	0.38
**Menstrual bleeding intensity** (reference: Moderate (>1 pad soaked in 3 hours))^**†**^					
Light (<1 pad soaked in 3 hours)	141 (13.3)	124 (87.9)	17 (12.1)	0.80 (0.47-1.39)	0.43
Moderate (>1 pad soaked in 3 hours)	748 (70.4)	639 (85.4)	109 (14.6)	–	–
Heavy (>1 pad soaked every 2 hours)	173 (16.3)	124 (71.7)	49 (28.3)	2.32 (1.57-3.42)	<0.001^d^
**Smoking status** (reference: never smoker)^**†**^					
Never smoker	687 (64.7)	590 (85.9)	97 (14.1)	–	–
Current smoker	340 (32.0)	264 (77.6)	76 (22.4)	1.75 (1.25-2.44)	<0.001^d^
Ex-smoker	35 (3.3)	33 (94.3)	2 (5.7)	0.37 (0.09-1.56)	0.16
**Physical exercise** (reference: no exercise)^**†**^					
No exercise	279 (26.3)	215 (77.1)	64 (22.9)	–	–
Occasional exercise	331 (31.2)	272 (82.2)	59 (17.8)	0.73 (0.49-1.08)	0.12
Regular exercise	452 (42.6)	400 (88.5)	52 (11.5)	0.44 (0.29-0.65)	<0.001^d^
**Sleep duration** (reference: normal sleep (7–9 h))^**†**^					
Normal sleep (7–9 h)	617 (58.1)	529 (85.7)	88 (14.3)	–	–
Short sleep (<7 h)	292 (27.5)	237 (81.2)	55 (18.8)	1.40 (0.96-2.02)	0.08
Long sleep (>9 h)	153 (14.4)	121 (79.1)	32 (20.9)	1.59 (1.01-2.49)	0.04^d^
**Meal skipping** (reference: low meal skipping)^**†**^					
Low meal skipping	658 (62.0)	563 (85.6)	95 (14.4)	–	0.02^d^
High meal skipping	404 (38.0)	324 (80.2)	80 (19.8)	1.46 (1.05-2.03)	
**Fast food consumption** (reference: Low fast-food consumption)					
Low fast-food consumption	615 (57.9)	520 (84.6)	95 (15.4)	–	0.29
High fast-food consumption	447 (42.1)	367 (82.1)	80 (17.9)	1.19 (0.86-1.65)	
**Bergen Social Media Addiction Scale (BSMAS)** ^**†**^					
BSMAS total score	16.16 ± 5.71	15.68 ± 5.64	18.59 ± 5.47	1.10 (1.06–1.13)	<0.001^d^
**War exposure** (reference: No reported war-related impact)^**†**^					
No reported war-related impact	766 (72.1)	654 (85.4)	112 (14.6)	–	0.01^d^
Any war-related impact	296 (27.9)	233 (78.7)	63 (21.3)	1.58 (1.12-2.22)	
**PSS-4 total score, mean ± SD** ^**†**^					
PSS-4 total score, mean ± SD	8.04 ± 2.10	7.89 ± 2.05	8.78 ± 2.19	1.24 (1.14–1.35)	<0.001^d^

***CI***, confidence interval; ***UOR***, unadjusted odds ratio.

a Percentages for the column.

b Percentages for the row.

c Univariate binary logistic regression was conducted to assess associations with PMDD.

d Statistically significant (*p* < 0.05).

† Retained in the final model.

**Note:** Collapsed variables were defined as follows: early undergraduate included freshman, first-year, and second-year students; advanced undergraduate/professional included third-, fourth-, fifth-, sixth-year, and PharmD students; postgraduate included Master’s/MBA and PhD/DBA students. Ever married included married, divorced, and widowed participants. Regular exercise included participants exercising at least 1–2 times per week. Low meal skipping included rarely/never and sometimes, while high meal skipping included occasionally and frequently. Low fast-food consumption included rarely/never and sometimes, while high fast-food consumption included occasionally and frequently. Any war-related impact included displacement from home, financial difficulties due to war, damage or destruction of workplace or home, or loss/injury of a family member.

**Table 7:** Multivariable binary logistic regression^a^ of factors associated with PMDD among female university students.

Predictors	UOR	B	SE	Wald	AOR	95% CI	*p*
**Constant**		−4.15	0.53	61.25	0.02		<0.001^b^
**Age** (reference: 18–20 years)							
21-24 years	0.89	0.008	0.27	0.001	1.008	0.59-1.72	0.98
25-30 years	0.52	−0.29	0.39	0.53	0.75	0.35-1.62	0.47
>30 years	0.30	−0.78	0.63	1.55	0.46	0.13-1.57	0.21
**Academic Specialization** (reference: non-medical)							
Medical/health-related	1.25	0.16	0.18	0.78	1.18	0.82-1.68	0.38
**Academic level** (reference: early undergraduate)							
Advanced undergraduate/professional	0.74	−0.03	0.27	0.01	0.97	0.57-1.65	0.92
Postgraduate	0.48	−0.07	0.40	0.03	0.93	0.43-2.03	0.85
**Menstrual cycle duration (days)** (reference: normal cycle (24–38 days))							
Frequent cycle (<24 days)	1.46	0.42	0.29	2.08	1.53	0.86-2.71	0.15
Infrequent cycle (>38 days)	2.93	0.70	0.57	1.50	2.01	0.66-6.16	0.22
**Menstrual bleeding intensity** (reference: moderate bleeding)							
Light (<1 pad soaked in 3 hours)	0.80	−0.20	0.29	0.48	0.82	0.46-1.45	0.49
Heavy (>1 pad soaked every 2 hours)	2.32	0.75	0.21	12.47	2.13	1.40-3.23	<0.001^b^
**Smoking status** (reference: never smoker)							
Current smoker	1.75	0.48	0.19	6.47	1.61	1.12-2.32	0.01^b^
Ex-smoker	0.37	−0.93	0.76	1.48	0.40	0.09-1.76	0.22
**Physical exercise** (reference: no exercise)							
Occasional exercise	0.73	−0.36	0.22	2.79	0.70	0.46-1.06	0.09
Regular exercise	0.44	−0.63	0.22	8.53	0.53	0.35-0.81	0.004^b^
**Sleep duration** (reference: normal sleep (7–9 h))							
Short sleep (<7 h)	1.40	0.17	0.20	0.72	1.19	0.80-1.78	0.40
Long sleep (>9 h)	1.59	0.15	0.25	0.35	1.16	0.71-1.89	0.55
**Meal skipping** (reference: low meal skipping)							
High meal skipping	1.46	0.20	0.18	1.21	1.22	0.86-1.73	0.27
**BSMAS total score**							
BSMAS total score	1.10	0.07	0.02	16.52	1.07	1.04-1.11	<0.001^b^
**War exposure** (reference: No reported war-related impact)							
Any war-related impact	1.58	0.36	0.19	3.67	1.44	0.99-2.08	0.06
**PSS-4 total score**							
PSS-4 total score	1.24	0.15	0.05	10.69	1.16	1.06-1.27	0.001^b^

***AOR***, adjusted odds ratio; ***B***, logistic regression coefficient; ***CI***, confidence interval; ***SE***, standard error; ***UOR***, unadjusted odds ratio; ***Wald***, Wald chi-square test that tests the null hypothesis.

a Multivariable binary logistic regression using the Enter method.

b Statistically significant (*p* < 0.05).

### Management practices for premenstrual syndrome

Among participants with moderate-to-severe PMS or PMDD (N = 672), 319 (47.5%) reported using at least one non-pharmacological strategy to manage symptoms. The most commonly reported approaches were sleep or rest (n = 229/319, 71.8%), increased water intake (n = 164/319, 51.4%), regular physical activity (n = 125/319, 39.2%), caffeine reduction (n = 90/319, 28.2%), hot baths (n = 87/319, 27.3%), and magnesium supplementation (n = 86/319, 27.0%). Herbal beverages and other complementary approaches were used less frequently. Most participants perceived lifestyle modifications as slightly effective (46.4%) or moderately effective (28.8%), whereas 5.6% considered them very effective and 19.1% reported no benefit.

Pharmacological treatment was used by 262 participants (n = 262/672, 39%). Pharmacists were the most frequently reported source of medication recommendation (n = 89/262, 34.1%), followed by family members or friends (n = 78/262, 29.9%) and self-initiated use (n = 67/262, 25.5%); physician recommendations accounted for 7.3%. Analgesics were used by 254 medication users (96.9%), most commonly ibuprofen, mefenamic acid, paracetamol, and diclofenac. Two-thirds of analgesic users reported use during every menstrual cycle, with a mean duration of 2.04 ± 0.91 days per cycle (Table 8).

**Table 8:** Utilization of analgesics for PMS management among participants (N = 254).

Information	Frequency (%)
**Utilized Analgesics**	
Ibuprofen	54 (21.3)
Mefenamic acid	49 (19.3)
Paracetamol	39 (15.4)
Diclofenac	36 (14.2)
Butyl scopolamine	18 (7.1)
Panadol women^®^ (Paracetamol, Hyoscine butyl bromide)	16 (6.3)
Gentle relief^®^ (Paracetamol, Ibuprofen, Famotidine)	12 (4.7)
Ketoprofen	10 (3.9)
Solpadeine^®^ (Paracetamol, Caffeine, Codeine)	9 (3.5)
Spasfon^®^ (Phloroglucinol hydrate, Trimethyl phloroglucinol)	2 (0.8)
Ketorolac	2 (0.8)
Flurbiprofen	1 (0.4)
Codeine	1 (0.4)
Diclocort b12^®^ (Diclofenac, Cyanocobalamin, Betamethasone)	1 (0.4)
Dolfein^®^ (Paracetamol, Caffeine, Codeine)	1 (0.4)
Muscerol^®^ 3 (Ibuprofen, Orphenadrine, Paracetamol)	1 (0.4)
Naproxen	1 (0.4)
Peladol^®^ (Paracetamol, Diclofenac, Caffeine)	1 (0.4)
**Frequency of Analgesic Utilization**	
Every cycle	171 (67.3)
Occasionally	53 (20.9)
Rarely	30 (11.8)
**Analgesic Utilization Duration (Days)**	
Mean ± standard deviation: 2.04 ± 0.91 (Range: 1–7)	

Antidepressants and hormonal therapies were used less frequently, by 6.1% and 1.5% of medication users, respectively. Overall, 45.8% of medication users considered pharmacological treatment very effective and 36.6% considered it moderately effective. Pain or cramps were the most frequently improved symptoms (92.7%), followed by back pain, general discomfort, and headache. Adverse effects were reported by 9.2% of medication users, most commonly stomach pain, sweating, nausea, and weight gain; one-third of those reporting adverse effects discontinued treatment.

## Discussion

This study has demonstrated the presence of a substantial burden of premenstrual symptoms among female university students in Lebanon. Nearly half of the participants screened positive for moderate-to-severe PMS, and 16.5% had symptoms consistent with PMDD based on the PSST. The most frequently reported physical manifestations were fatigue, depressed mood, and food cravings, with more than one-third of participants reporting PMS-related academic absenteeism. Heavy menstrual bleeding, current smoking, meal skipping, frequent fast-food consumption, higher social media addiction scores, and higher perceived stress scores were independently associated with moderate-to-severe PMS/PMDD. Although lifestyle modifications were used by almost half of participants with clinically significant symptoms, pharmacological management was reported by 39.0%, mainly through analgesic use, and professional consultation was limited.

The observed prevalence was higher than the estimated global prevalence of approximately 20–30% [33] but comparable to that reported in Saudi Arabia (47.1%) [6]. Higher estimates have been reported in other regional studies and in a previous Lebanese study [3,10,11,14,36]. Such variation may reflect differences in diagnostic instruments, severity thresholds, sample composition, and exclusion criteria. The present study used the PSST, which incorporates both symptom severity and functional impairment, whereas other studies used alternative assessment methods [10,14].

Despite the relatively lower prevalence compared with some regional reports, the prevalence observed in the current study remains relatively high, possibly influenced by the young age of participants, as nearly half were between 18 and 20 years, an age group potentially more susceptible to hormonal fluctuations [35,37].

Regarding PMDD, the current study demonstrated a prevalence of 16.5%, which is higher than the estimated global prevalence of approximately 3–8%, and also higher than that reported in Iran (7.1%) and Jordan (7.7%). The relatively elevated PMDD prevalence may suggest that Lebanese females experience a greater burden of severe emotional and functional symptoms rather than milder PMS manifestations. One possible explanation may be chronic psychosocial stressors in Lebanon, including economic instability, political uncertainty, and war-related stress exposure [29,38–40]. Additionally, limited access to mental health support and suboptimal coping strategies may further contribute to the observed burden.

Physical symptoms, fatigue, depressed mood, irritability, food cravings, and concentration difficulties were common in this study, consistent with findings from Palestinian and Lebanese studies [12,14]. Hormonal fluctuations during the late luteal phase may influence pain perception, inflammatory mediators, and prostaglandin activity, contributing to abdominal pain, headache, breast tenderness, and bloating [41,42]. Food cravings may also be related to luteal-phase serotonin fluctuations [42,43].

The current study further demonstrated that PMS symptoms significantly interfered with several aspects of daily functioning, particularly home responsibilities, social activities, and academic productivity. Similar findings were reported in Saudi Arabia, where PMS was associated with poor concentration, reduced attendance, decreased performance of daily household activities, and reduced participation in social activities [6]. Likewise, an Afghan study suggested that PMS may substantially impair several aspects of females’ wellbeing, including daily productivity, interpersonal interactions, emotional wellbeing, and perception of self-worth [5]. These findings emphasize that PMS is not merely a physiological condition, but also a significant psychosocial and functional burden among young females. The greatest interference with home responsibilities may partially reflect traditional gender roles and domestic expectations placed on females within Lebanese society [44].

The substantial impact on academic performance is particularly concerning given that more than one-third of participants (37.2%) reported academic absenteeism directly attributable to PMS symptoms. High absenteeism rates may suggest inadequate symptom control, delayed healthcare-seeking behavior, and reliance on self-management strategies rather than professional medical consultation. Importantly, the actual academic burden of PMS may be underestimated, as many females may continue attending classes despite reduced concentration and productivity. These findings highlight the need for increased awareness, early recognition, and appropriate management strategies among female university students.

The current study revealed several independent predictors significantly associated with moderate-to-severe PMS as well as PMDD, including heavy menstrual bleeding, smoking, low physical activity, social media addiction, psychological stress, unhealthy dietary intake, and skipping meals. Heavy menstrual blood flow was significantly associated with moderate-to-severe PMS and PMDD in the current study. This finding is consistent with previous studies with a statistically significant p-value [45,46], although some contradictory studies reported no significant association between menstrual blood flow and PMS or PMDD [18,47]. This relationship may be explained by increased endometrial prostaglandin production, which intensifies uterine contractions and may also contribute to systemic symptoms [48].

A significant association was observed between current smoking and the occurrence of moderate-to-severe PMS and PMDD. This finding is supported by other studies conducted in Spain (OR = 1.78 for PMS, OR = 2.92 for PMDD) [5] and Turkey (OR = 2.36 for PMS) [17]. However, contradictory findings have also been reported, showing no significant association between smoking and PMS. This association can be interpreted by multiple biological mechanisms, including mood and hormonal dysregulation via dopaminergic and serotonergic pathways, increased sympathetic nervous system activity, and disruption of the hypothalamic–pituitary–gonadal (HPG) axis [17]. Therefore, smoking cessation should be strongly recommended for all women, not only for general health benefits but also as a potential strategy to reduce the risk and severity of PMS and PMDD symptoms.

A study conducted in India provided evidence that physical activity can effectively reduce the severity of premenstrual disorders, including PMS and PMDD [49]. However, a contradictory study found no significant association between regular physical activity and the occurrence of PMS or PMDD (p = 0.357) [46]. In the present study, regular physical exercise was not significantly associated with improvement in PMS symptoms, although a significant association was observed with improvement in PMDD symptoms. This finding suggests that physical activity may have a greater impact on the more severe psychological and emotional manifestations characteristic of PMDD rather than PMS. Interestingly, physical activity can increase endorphin levels, help regulate hormonal balance, and stimulate anti-inflammatory processes, all of which may contribute to symptoms alleviation [50]. Universities should consider promoting accessible physical activity opportunities for female students.

High fast-food consumption and meal skipping were independently associated with moderate-to-severe PMS, consistent with previous studies [19,20]. Unhealthy dietary patterns may increase inflammatory burden, while meal skipping may contribute to inadequate intake of micronutrients involved in hormonal regulation and neurotransmitter synthesis [20,51]. University health-promotion initiatives should encourage regular meals and healthier dietary choices.

Higher perceived stress was associated with moderate-to-severe PMS and PMDD, consistent with findings from India, Jordan, and Ethiopia [45,52,53]. Stress may intensify symptoms through cortisol-related disruption of hormonal and neurotransmitter regulation [2].

Higher social media addiction scores were also associated with moderate-to-severe PMS and PMDD, consistent with Turkish and Iranian findings [54]. Excessive social media use may contribute to emotional sensitivity, mood instability, and psychological distress.

Although lifestyle modifications are recommended as first-line treatment for mild to moderate PMS, fewer than half of participants (47.5%) reported using them, indicating underutilization. Among those who adopted these strategies, regular physical exercise was one of the most common approaches. Exercise may alleviate PMS symptoms by improving mood, reducing stress, and decreasing overall symptom severity through endorphin release and reduced cortisol levels [55,56]. Evidence from previous studies and systematic reviews supports the effectiveness of exercise, sleep optimization, and relaxation techniques in reducing premenstrual symptoms [16,25,]. Evidence from previous studies and systematic reviews supports the effectiveness of exercise, sleep optimization, and relaxation techniques in reducing premenstrual symptoms [16,25,]. Together, these findings highlight the importance of integrating lifestyle-based approaches, particularly exercise and sleep hygiene, into PMS management strategies and support the implementation of wellness initiatives within university settings.

Cravings for chocolate, pastries, and other sweet foods commonly occur during the 7–10 days preceding menstruation; however, frequent consumption of high-calorie, fatty, sugary, and salty foods may worsen PMS symptoms. Consequently, maintaining a balanced and healthy diet may help reduce symptom severity [16,26]. Concerning supplements, in this study, magnesium supplementation was used by 27% of participants. Magnesium plays a vital role in numerous biochemical processes and has been associated with improvements in mood-related and physical symptoms that overlap with PMS. Evidence suggests that supplementation with 250 mg of magnesium daily, particularly when combined with vitamin B6 and continued for at least two months, may effectively alleviate PMS symptoms [57,58]. Additionally, herbal products such as chamomile, anise, ginger, and Valeriana may provide benefit through their antioxidant, anti-inflammatory, analgesic, sedative, and muscle-relaxant properties, which may help reduce the oxidative stress and inflammation implicated in PMS [59].

Women with moderate-to-severe PMS or PMDD symptoms, or symptoms that do not improve with non-medical treatments, may need medication. Since these conditions are linked to brain chemicals, hormones, and the reproductive hormone system, treatments focus on both neurotransmitters in the brain and the hypothalamus-pituitary-ovarian axis [22,23]. According to ACOG guidelines, medication choice depends on the patient’s main symptoms, such as physical symptoms, mood-related symptoms, and the need for contraception.

Analgesics were the most commonly used pharmacological treatments, reflecting the high burden of pain-related symptoms. This pattern is consistent with Saudi findings showing frequent self-management with NSAIDs. NSAIDs are effective for menstrual pain because they reduce prostaglandin-mediated uterine contractions and are most effective when initiated shortly before menstruation [60].

Antidepressant use for the management of PMS was relatively uncommon in the present study, with only 6.1% of participants reporting their use. The low use of antidepressants may reflect limited awareness of evidence-based PMS and PMDD treatments, concerns about adverse effects, stigma surrounding psychotropic medications, or a preference for analgesics and non-pharmacological approaches. SSRIs are well-established pharmacological options for moderate-to-severe PMS and PMDD, particularly when emotional or behavioral symptoms predominate [15,22,23,61]. Unlike their use in major depressive disorders, SSRIs may produce a relatively rapid improvement in premenstrual symptoms and can be prescribed using flexible regimens, including continuous or luteal-phase dosing [22,23]. Findings from a Cochrane review indicate that SSRIs are effective for PMS and PMDD when administered either continuously or intermittently [62]. Therefore, the low antidepressant use observed in this study should not be interpreted as absence of need, but rather as a potential gap in awareness, counseling, help-seeking behavior, or access to appropriate care. These findings highlight the importance of university-based education and referral pathways, particularly for students experiencing severe mood-related premenstrual symptoms.

Hormonal therapies were rarely used, possibly because of concerns regarding adverse effects, medical supervision, and sociocultural barriers to contraceptive use among unmarried women. Combined oral contraceptives may be appropriate when physical symptoms predominate or contraception is desired, particularly formulations containing drospirenone and ethinylestradiol [22,23,63,64].

Regarding management seeking, female participants were generally reluctant to seek professional medical advice for PMS and tended to rely more on informal support networks, such as family and friends. Pharmacists were the most common source of medication recommendations, followed by family or friends, and self-decision, while physicians were the least consulted. These findings emphasize the important role of community pharmacists as accessible healthcare providers, as well as the widespread reliance on self-medication and informal guidance. The low rate of physician involvement may reflect barriers to healthcare access or the perception that PMS can be managed without professional care. This perception is likely influenced by cultural and societal beliefs that menstrual pain is a normal physiological experience, encouraging women to adopt self-care strategies rather than seek medical consultation.

### Study limitations

Several limitations should be considered when interpreting the findings of this study. First, the PSST is a subjective, self-reported screening instrument rather than an objective diagnostic tool. Consequently, the reported prevalence rates of PMS and PMDD may not fully reflect clinically confirmed diagnoses. Second, the use of a self-administered questionnaire introduces multiple potential biases including, participants may have overreported or underreported symptoms based on subjective perception, voluntary participation may have led to selection bias, as females experiencing more severe symptoms may have been more interested in participating in the study, and despite anonymity, the sensitive nature of menstrual and emotional symptoms may have led some participants to underreport. In addition, the cross-sectional design and reliance on retrospective self-reporting may have increased the possibility of recall bias. Self-reported medical and gynecological conditions used to determine eligibility may also have resulted in misclassification of some participants.

The study was conducted among female students at a single university in Lebanon. Although data were collected across all four campuses of Beirut Arab University and included students from diverse geographical regions, the use of convenience sampling within one institution may limit the generalizability of the findings to all female university students in Lebanon. Furthermore, because Beirut Arab University is a private institution, the study population may not fully represent students enrolled in public universities or other higher education institutions in Lebanon. Differences in socioeconomic characteristics and financial stressors between students attending public and private universities may influence the prevalence and severity of PMS and PMDD and should therefore be considered when interpreting the findings. In addition, the unequal distribution of participants across faculties and academic fields may have affected sample representativeness, as health-related students may have greater awareness of PMS symptoms and management options than non-medical students. Moreover, the exclusion of participants with gynecological, endocrine, psychiatric, pregnancy-related, or hormonal medication-related conditions may have improved internal validity and homogeneity but may also limit the generalizability of the findings to the wider population of reproductive-aged university women.

Furthermore, some potential confounding factors and covariates that may influence PMS severity were not extensively assessed. For example, factors such as family history of PMS, caffeine intake, detailed dietary patterns, medication adherence, sleep quality, and prior healthcare-seeking behavior were not comprehensively evaluated. Additionally, the perceived effectiveness of pharmacological and non-pharmacological management practices was based on participants’ subjective assessment rather than objective pre- and post-intervention symptom measurement. Finally, the cross-sectional nature of the study limits the ability to establish causal relationships between associated factors and PMS or PMDD, although the findings may help generate hypotheses for future longitudinal research. Future multicenter studies involving students from diverse educational institutions, including both public and private universities, and different geographic regions across Lebanon are warranted to provide more representative national estimates and enhance the generalizability of the findings.

## Conclusions

This study showed that PMS was common among female university students in Lebanon, with a substantial proportion experiencing moderate-to-severe symptoms and symptoms consistent with PMDD. Premenstrual symptoms were not only frequent but also functionally relevant, as they interfered with daily activities and contributed to academic absenteeism among a considerable proportion of participants. Physical symptoms, fatigue, depressed mood or hopelessness, and food cravings were among the most commonly reported moderate-to-severe symptoms.

Several factors were independently associated with moderate-to-severe PMS, including heavy menstrual bleeding, current smoking, frequent meal skipping, high fast-food consumption, higher social media addiction scores, and higher perceived stress scores. These findings highlight the multifactorial nature of PMS and suggest that both biological and modifiable lifestyle as well as psychosocial factors may contribute to symptom burden among university students. The findings also emphasize the importance of addressing stress, unhealthy dietary behaviors, smoking, and problematic social media use as part of comprehensive PMS awareness and management strategies.

Given the high burden observed, universities should consider implementing targeted educational and counseling interventions to improve students’ awareness of PMS, encourage appropriate health-seeking behavior, and promote evidence-based symptom management. Future studies using prospective symptom tracking and multicenter designs are warranted to confirm these findings and further explore the effectiveness of different PMS management approaches among university students in Lebanon.

## Supporting information

S1 Appendix Study questionnaire. Self-administered questionnaire used to collect sociodemographic, menstrual, lifestyle, psychosocial, and premenstrual syndrome management-related data from female university students in Lebanon.
(PDF)

S1 Data De-identified study dataset. De-identified individual-level dataset underlying the findings reported in this study.
(XLSX)

S1 Table Premenstrual symptoms (N = 1,062). (DOCX)

S2 Table Interference with life (N = 1,062). (DOCX)
