# Supplementary material for: Prevalence, risk factors, and management practices of premenstrual syndrome among female university students in Lebanon: An observational cross-sectional study
Source: PLoS One. 2026 Jul 27;21(7):e0354807. doi: 10.1371/journal.pone.0354807 (PMC13405303; doi:10.1371/journal.pone.0354807)
Supplement: S1 Appendix — Self-administered questionnaire used to collect sociodemographic, menstrual, lifestyle, psychosocial, and premenstrual syndrome management-related data from female university students in Lebanon. (PDF) [file pone.0354807.s001.pdf]

Supplementary Appendix: Questionnaire

Questionnaire on Premenstrual Symptoms, Lifestyle, and Management

Dear Lady,

The following study aims to investigate the prevalence, risk factors, and management practices of **premenstrual syndrome (PMS)** among female university students. PMS is a group of **physical, emotional, and behavioral** symptoms such as **mood changes, fatigue, and bloating** that occur in the **5 days before your period** and usually **improve shortly after the onset of the menstrual period**. The results of this study will help us to formulate targeted educational sessions to improve proper management of PMS.

If you are a university student, you are invited to participate in this study by filling out a questionnaire that takes approximately 10-15 minutes to be completed. Your participation is important to us.

Informed consent

Your participation in this survey is **voluntary**; you have the right not to participate or withdraw at any time. This survey is intended for research purposes and your responses will be kept **confidential.**

I voluntarily agree to participate in this research study. I have had the purpose and nature of the study explained to me in writing and I have had the opportunity to ask questions about the study. I understand that even if I agree to participate now, I can withdraw at any time or refuse to answer any question without any consequences of any kind and I understand that all the information I provided for this study will be treated confidentially. If you have questions or concerns, you can ask questions about this research study by talking to the researcher(s).

Questionnaire form

**Sociodemographic information**
**1. Age:** ___ years<br><br>**2. Faculty:**<br>○ Agricultural & Food Sciences ○Business Administration ○Dentistry<br>○Education ○Engineering & Architecture ○Health Sciences<br>○Human Sciences ○Language & Translation ○Law & Political Sciences<br>○Medicine ○Pharmacy ○Religious Studies<br>○Sciences ○Technology ○Other: ____<br><br>**3. Academic level:**<br>○Freshman ○1st ○2nd ○3rd ○4th ○5th ○6th ○PharmD ○Master's/MBA<br>○PhD/DBA<br><br>**4. Marital status:**<br>○Single/engaged ○Divorced<br>○Married ○Widowed<br>**5. Weight:** _____ kg<br>**6. Height:** _____ cm

**Menstrual history**
**7. At what age did you have your first menstrual period.** ____ years<br><br>**8. On average, how many days are there between the first day of one period and the first day of the next period?** ____ days<br><br>**9. On average, how many days does your menstrual bleeding last?** ____ days<br><br>**10. How heavy is your period?**<br>○Light (<1 pad soaked in 3 hours)<br>○Moderate (>1 pad soaked in 3 hours)<br>○Heavy (>1 pad soaked every 2 hours)

Medical and medication history
**11. Do you think that you may be suffering from PMS?**<br>○Yes ○No<br><br>**12. Are you currently pregnant or breastfeeding?**<br>○Yes ○No<br><br>**13. Do you have any of the following:**<br>□Thyroid disorders<br>□Polycystic ovary syndrome (PCOS)<br>□Do not have a period for 3 months or more (amenorrhea)<br>□Tissue grows outside the uterus (endometriosis)<br>□Small harmless masses in uterus (uterine fibroids)<br>□Small benign growths on the lining of the uterus (polyps)<br>□Chronic pelvic inflammation<br>□Diagnosed mental health conditions (e.g., depression, anxiety)<br>□Other gynecological (women-related) condition, please specify: ____<br>□None of the above<br><br>**14. Are you currently taking: (Select all that apply)**<br>□Hormonal therapies (e.g., birth control pills)<br>□Medication for anxiety, depression, or another mental health condition<br>□None of the above

Premenstrual symptoms

Part 1: Symptoms

15. Do you experience any of the following symptoms before your period that improve within a few days after bleeding starts?

Please rate each symptom:

Symptom	Not at all	Mild	Moderate	Severe
**A.** Anger / irritability	○	○	○	○
**B.** Anxiety / tension	○	○	○	○
**C.** Tearful / increased sensitivity to rejection	○	○	○	○
**D.** Depressed mood / hopelessness	○	○	○	○
**E.** Decreased interest in work activities	○	○	○	○
**F.** Decreased interest in home activities	○	○	○	○
**G.** Decreased interest in social activities	○	○	○	○
**H.** Difficulty concentrating	○	○	○	○
**I.** Fatigue / lack of energy	○	○	○	○
**J.** Overeating / food cravings	○	○	○	○
**K.** Insomnia (difficulty to sleep)	○	○	○	○
**L.** Hypersomnia (needing more sleep)	○	○	○	○
**M.** Feeling overwhelmed or out of control	○	○	○	○
**N.** Physical symptoms (breast pain, headache, muscle pain, swollen stomach, weight gain)	○	○	○	○

Part 2: Interference with life

Have your symptoms, as listed above, interfered with…

Area	Not at all	Mild	Moderate	Severe
**A.** Study efficiency or productivity	○	○	○	○
**B.** Relationships with classmates	○	○	○	○
**C.** Relationships with family	○	○	○	○
**D.** Social life activities	○	○	○	○
**E.** Home responsibilities	○	○	○	○

16. Have your symptoms caused you to miss classes?

○ Yes ○No

Lifestyle habits

17. What is your smoking status?

○Current Smoker ○Ex-smoker ○Never Smoker

If current smoker, what do you use? (Select all that apply)

○Cigarette ○Electronic cigarettes/vape/IQOS

○Hookah (nargila)

If you smoke cigarettes, how many **cigarettes** do you smoke **per day?**

○ 5 or less ○6 -10 ○11-20 ○21-30 ○31 or more

If you smoke hookah, how many **hookah sessions** do you smoke **per day**?

○1 ○2 ○3 ○4 ○5 ○6 or more

18. How often do you exercise?

○ Daily ○ 3–4 times per week ○ Occasionally

○ 5–6 times per week ○ 1–2 times per week ○ I do not exercise

19. How many hours do you sleep each night? ____ hours

20. Social Media Addiction Scale
Please select the best answer

Statement	Very rarely	Rarely	Sometimes	Often	Very often
**A.** You spend a lot of time thinking about social media or planning how to use it.	○	○	○	○	○
**B.** You feel an urge to use social media more and more.	○	○	○	○	○
**C.** You use social media in order to forget about personal problems.	○	○	○	○	○
**D.** You have tried to cut down on the use of social media without success.	○	○	○	○	○
**E.** You become restless or troubled if you are prohibited from using social media.	○	○	○	○	○

**F.** You use social media so much that it has had a negative impact on your job/studies.	○	○	○	○	○

21. How often do you skip meals during the day?
○Frequently ○Occasionally ○Sometimes ○Rarely/ never

22. How often do you eat fast food?
○Frequently ○Occasionally ○Sometimes ○Rarely/ never

Psychological conditions

23. Stress Scale
Please select the best answer

In the last month,	Never	Almost Never	Some times	Fairly Often	Very Often
A. How often have you felt that you were **unable** to **control** the important things in your life?	○	○	○	○	○
B. How often have you felt **confident** about your ability to **handle** your personal problems?	○	○	○	○	○
C. How often have you felt that things were **going your way**?	○	○	○	○	○
D. How often have you felt **overwhelmed** by too many things?	○	○	○	○	○

Lifestyle modifications for PMS Management

24. Do you follow any lifestyle changes to manage your symptoms?

○Yes ○No

If yes, which ones? (Select all that apply)

→Relaxing therapies:

☐Sleep/rest ☐Yoga ☐Hot bath

☐Regular exercise (walking, running, gym workouts...)

→Diet changes:

☐Reduce salt ☐Reduce Caffeine ☐Increase water intake ☐Drink anise tea

☐Drink chamomile tea ☐Drink Ginger tea ☐Drink cinnamon tea

→ Herbal remedies/supplements:

☐Ashwagandha ☐Magnesium ☐Normocalm ☐Other: ______

25. How effective are lifestyle changes in reducing your symptoms?

○Not effective ○Moderately effective

○Slightly effective ○Very effective

Medications used for PMS Management

26. Which types of medications do you use to manage your premenstrual symptoms? (Select all that apply)

☐Pain relievers ☐Medications for depression/mood changes ☐Hormonal therapy

☐None ☐Other, specify: ___

If your answer to the previous question is "None," you have completed the questionnaire. Thank you for your time.
However, if you use any of the above medications, please continue. The following sections will open based on the medication you take.

27. What is the source of the recommendation? (Select all that apply)

- Prescribed by a physician
- Prescribed by a pharmacist
- Recommended by a nurse
- Family or friends

- Social media
- Self-decision/ without prescription

Use of pain killers for PMS Management

28. Which pain reliever do you use most often?

○Advil ○Buscopan ○Captain ○Cataflam ○Diclogesic ○Divido
○Doloraz ○Epardex ○Gentle relief ○Ketolac ○Nopain ○Olfen
○Profinal ○Pangesic ○Panadol ○Panadol women ○Ponstan forte ○Proxen
○Profenid ○Rofenac-D ○Remethan ○Scopinal ○Solpadeine ○Voltfast
○Voltaren ○Voldic-K ○Other: _____

29. How often do you use pain relievers before your period?

○Every cycle ○Occasionally ○Rarely

30. For how many days per cycle do you usually take them? ___ days

Use of medications for mood symptoms in PMS management

31. Which medication for depression do you use?

○Apo-escitalopram ○Apo-Paroxetine ○Cipralex ○Cipram ○Citoles
○Deanxit ○Deprevix ○Deprine ○Deprox
○Duloxetine
○Effexor ○Escitalopram biogaran ○Fluoxone divule ○Lexotanil
○Lorazepam
○Loxyt ○Neanxetin ○Parlotin ○Paxera
○Proxetin ○Prozac ○Prylex
○Seroxat ○Sertine ○ Sertra tad ○Solotik
○Venlax ○Xanax ○Zoloft ○Other: _____

32. How do you usually take the medication?

○Daily throughout the cycle ○Only before the period ○Only when symptoms are severe

Use of hormonal therapy

33. Which hormonal therapy do you use?

○Cerazette ○Diane ‘35’ ○Duphaston ○Marvelon ○Microgynon ○Primolut nor
○Progest ○Progylton ○Yasmin ○Yaz ○Other: ______

34. How do you take the medication?

○I take them daily without stopping ○I take them for 21days and 7 days without or empty pills
○I don’t take them regularly

Assessment

35. How effective are the medications in reducing your symptoms?

○Not effective ○Moderately effective
○Slightly effective ○Very effective

36. Which symptoms improve the most? (Select all that apply)

☐Pain/cramps ☐Mood changes ☐Bloating ☐Irregular cycles
☐Irritability ☐Anxiety ☐Depression ☐Multiple symptoms
☐Headache ☐Back pain ☐General discomfort ☐Leg/hand swelling
☐Sleep disturbances ☐Other: ______

37. Have you experienced side effects from the medications?

○Yes ○No

If yes, which ones? (Select all that apply)

☐Weight gain ☐Mood changes ☐Headaches ☐Nausea ☐Breast pain
☐Irregular bleeding ☐Insomnia ☐Drowsiness ☐Sexual dysfunction
☐Hair loss ☐Increased appetite ☐Decreased appetite
☐Stomach pain ☐Sweating ☐Sleep changes
☐Irregular spotting ☐Acne ☐Other: ______

38. Did side effects lead you to stop treatment?

○Yes ○No
