# Supplementary material for: Prevalence, risk factors, and management practices of premenstrual syndrome among female university students in Lebanon: An observational cross-sectional study
Source: PLoS One. 2026 Jul 27;21(7):e0354807. doi: 10.1371/journal.pone.0354807 (PMC13405303; doi:10.1371/journal.pone.0354807)
Supplement: S1 Table — (DOCX) [file pone.0354807.s003.docx]

**S1 Table.** Premenstrual symptoms (N = 1,062)

| Do you experience any of the following symptoms before your period that improve within a few days after bleeding starts? | | | | | |
| --- | --- | --- | --- | --- | --- |
| **Symptom** | **Not at all**  **n (%)** | **Mild**  **n (%)** | **Moderate**  **n (%)** | **Severe**  **n (%)** | **Mean ± SD** |
| Q1. Anger / irritability | 140 (13.2) | 277 (26.1) | 437 (41.1) | 208 (19.6) | 1.67 ± 0.93 |
| Q2. Anxiety / tension | 139 (13.1) | 319 (30) | 427 (40.2) | 177 (16.7) | 1.6 ± 0.91 |
| Q3. Tearful / increased sensitivity to rejection | 137 (12.9) | 272 (25.6) | 368 (34.7) | 285 (26.8) | 1.75 ± 0.99 |
| Q4. Depressed mood / hopelessness | 83 (7.8) | 259 (24.4) | 470 (44.3) | 250 (23.5) | 1.84 ± 0.87 |
| Q5. Decreased interest in work activities | 138 (13) | 352 (33.1) | 402 (37.9) | 170 (16) | 1.57 ± 0.9 |
| Q6. Decreased interest in home activities | 144 (13.6) | 306 (28.8) | 391 (36.8) | 221 (20.8) | 1.65 ± 0.95 |
| Q7. Decreased interest in social activities | 147 (13.8) | 342 (32.2) | 426 (40.1) | 147 (13.8) | 1.54 ± 0.89 |
| Q8. Difficulty concentrating | 277 (26.1) | 412 (38.8) | 275 (25.9) | 98 (9.2) | 1.18 ± 0.92 |
| Q9. Fatigue / lack of energy | 87 (8.2) | 245 (23.1) | 448 (42.2) | 282 (26.6) | 1.87 ± 0.89 |
| Q10. Overeating / food cravings | 115 (10.8) | 244 (23) | 411 (38.7) | 292 (27.5) | 1.83 ± 0.95 |
| Q11. Insomnia | 509 (47.9) | 331 (31.2) | 150 (14.1) | 72 (6.8) | 0.8 ± 0.92 |
| Q12. Hypersomnia (needing more sleep) | 304 (28.6) | 287 (27) | 317 (29.8) | 154 (14.5) | 1.3 ± 1.03 |
| Q13. Feeling overwhelmed or out of control | 237 (22.3) | 333 (31.4) | 314 (29.6) | 178 (16.8) | 1.41 ± 1.01 |
| Q14. Physical symptoms (breast pain, headache, muscle pain, swollen stomach, weight gain) | 81 (7.6) | 241 (22.7) | 397 (37.4) | 343 (32.3) | 1.94 ± 0.92 |
