# Supplementary material for: Prevalence, risk factors, and management practices of premenstrual syndrome among female university students in Lebanon: An observational cross-sectional study
Source: PLoS One. 2026 Jul 27;21(7):e0354807. doi: 10.1371/journal.pone.0354807 (PMC13405303; doi:10.1371/journal.pone.0354807)
Supplement: S2 Table — (DOCX) [file pone.0354807.s004.docx]

**S2 Table.** Interference with life (N = 1,062)

| Have your symptoms, as listed above, interfered with… | | | | | |
| --- | --- | --- | --- | --- | --- |
| **Area** | **Not at all**  **n (%)** | **Mild**  **n (%)** | **Moderate**  **n (%)** | **Severe**  **n (%)** | **Mean ± SD** |
| Q15. Study efficiency or productivity | 199 (18.7) | 404 (38) | 373 (35.1) | 86 (8.1) | 1.33 ± 0.87 |
| Q16. Relationships with colleagues | 356 (33.5) | 400 (37.7) | 260 (24.5) | 46 (4.3) | 1 ± 0.86 |
| Q17. Relationships with family | 245 (23.1) | 380 (35.8) | 346 (32.6) | 91 (8.6) | 1.27 ± 0.91 |
| Q18.Social life activities | 181 (17) | 407 (38.3) | 386 (36.3) | 88 (8.3) | 1.36 ± 0.85 |
| Q19. Home responsibilities | 197 (18.5) | 329 (31) | 361 (34) | 175 (16.5) | 1.48 ± 0.97 |
