# Supplementary material for: Prevalence, risk factors, and management practices of premenstrual syndrome among female university students in Lebanon: An observational cross-sectional study
Source: PLoS One. 2026 Jul 27;21(7):e0354807. doi: 10.1371/journal.pone.0354807 (PMC13405303; doi:10.1371/journal.pone.0354807)
Supplement: Attachment — Submitted filename: 2-Responses to reviewers Comments PO RI.docx [file pone.0354807.s007.docx]

**Dear Prof. Jad El Masri,**

Academic Editor

PLOS ONE

Submission ID #: PONE-D-26-25846

Thank you for giving us the opportunity to submit a revised draft of our manuscript titled “**Prevalence, Risk Factors, and Management Practices of Premenstrual Syndrome among Female University Students in Lebanon: An Observational Cross-Sectional Study**” to PLOS One. We appreciate the time and effort that you and the reviewers have dedicated to providing valuable feedback on our manuscript. We are grateful to your insightful comments. We have been able to incorporate changes to reflect all of the suggestions you and the reviewers have provided. Those changes are highlighted within the manuscript.

Please see below, in **blue**, for point-by-point responses to the comments and concerns

**Editor’s comments:**

1- Please ensure that your manuscript meets PLOS ONE's style requirements, including those for file naming.

**Author’s response:** Thank you for this important reminder. We carefully reviewed the PLOS ONE style requirements and reformatted the manuscript accordingly. Specifically, we revised the title page, author affiliations, section headings, page numbering, continuous line numbering, spacing, table formatting, figure citations and captions, and reference citation style to align with the journal’s guidelines. All main headings and subheadings were revised to sentence case, and tables were retained as editable tables and placed directly after their first citation in the text. Figure files were prepared for separate upload using the required naming conventions, while their captions were retained in the manuscript. We also reviewed the Supporting Information labels and file names to ensure consistency with PLOS ONE requirements.

2- In the online submission form you indicate that your data is not available for proprietary reasons and have provided a contact point for accessing this data. Please note that your current contact point is a co-author on this manuscript. According to our Data Policy, the contact point must not be an author on the manuscript and must be an institutional contact, ideally not an individual. Please revise your data statement to a non-author institutional point of contact, such as a data access or ethics committee, and send this to us via return email. Please also include contact information for the third party organization, and please include the full citation of where the data can be found.

**Author’s response:**

Thank you for this important comment. We agree that the Data Availability Statement should identify a non-author institutional contact point and clearly explain the reason for restricted data access. The individual-level dataset contains sensitive information related to participants’ menstrual health, psychosocial characteristics, lifestyle behaviors, and health-related practices. Although no direct identifiers were collected, public sharing of the full dataset may pose a risk of participant re-identification because of the sensitive nature and combination of variables collected. Accordingly, we revised the Data Availability Statement to remove the co-author contact point and to designate the Institutional Review Board of Beirut Arab University as the non-author institutional contact responsible for reviewing requests for access to de-identified data. We also clarified that the data were collected directly by the research team at Beirut Arab University and were not obtained from, owned by, or deposited with a third-party organization. Therefore, no third-party data-source citation, repository DOI, or external dataset citation is applicable. The revised statement is as follows:

**Availability of data and materials:**

“The individual-level data underlying this study contain sensitive information related to menstrual health, psychosocial characteristics, lifestyle behaviors, and health-related practices. Although direct identifiers were not collected, public sharing of the full dataset may pose a risk of participant re-identification because of the sensitive nature and combination of variables collected. The data were collected directly by the research team at Beirut Arab University and were not obtained from, owned by, or deposited with a third-party organization. Therefore, no external dataset citation, repository DOI, or third-party data-source citation is applicable. De-identified data may be made available to qualified researchers for legitimate scientific purposes, subject to review and approval by the Institutional Review Board of Beirut Arab University. Requests should be submitted to the Institutional Review Board, Beirut Arab University, Beirut, Lebanon, at [irb@bau.edu.lb](mailto:irb@bau.edu.lb). The Institutional Review Board will assess whether the proposed use is consistent with the original ethical approval, informed-consent conditions, and participant confidentiality protections.”

3- Your ethics statement should only appear in the Methods section of your manuscript. If your ethics statement is written in any section besides the Methods, please move it to the Methods section and delete it from any other section. Please ensure that your ethics statement is included in your manuscript, as the ethics statement entered into the online submission form will not be published alongside your manuscript.

**Author’s response:**

Thank you for this important comment. We confirm that the ethics statement has been retained within the Methods section under the subsection “Ethical considerations.” The ethics statement was reviewed to ensure that it includes the ethical approval source, approval date, informed-consent procedure, voluntary nature of participation, and confidentiality safeguards. Any duplicate ethics statement appearing elsewhere in the manuscript, including the end-matter declarations, has been removed.

4- Please include captions for your Supporting Information files at the end of your manuscript, and update any in-text citations to match accordingly.

**Author’s response:**

Thank you for this important comment. We have added a Supporting Information section at the end of the manuscript and included a caption for the supplementary appendix containing the full study questionnaire. The supporting file has been labelled as S1 Appendix, and the in-text citation in the Methods section has been revised accordingly. The revised citation and caption are as follows:

**Questionnaire development and structure**

“A self-administered questionnaire was developed by the research team following a review of relevant literature [5,6,8,35]. It consisted of 39 closed-ended questions covering sociodemographic characteristics, menstrual history, premenstrual symptoms, lifestyle and psychosocial factors, and PMS management practices. The questionnaire was administered in English, and the PSST, BSMAS, and PSS-4 were used in their original English versions. The full questionnaire is provided in **S1 Appendix**.”

**Supporting information**

“**S1 Appendix.** Study questionnaire. Self-administered questionnaire used to collect sociodemographic, menstrual, lifestyle, psychosocial, war-exposure, and premenstrual syndrome management-related data from female university students in Lebanon.”

5- If the reviewer comments include a recommendation to cite specific previously published works, please review and evaluate these publications to determine whether they are relevant and should be cited. There is no requirement to cite these works unless the editor has indicated otherwise.

**Author’s response:**

Thank you for this clarification. We carefully reviewed all references suggested by the reviewers and evaluated their relevance to the study background, methodology, and interpretation of findings. We included only those publications that were directly relevant to the revised manuscript. References that were not directly applicable to the scope or objectives of the present study were not added.

**Additional Editor Comments:**

1- Most sections of the manuscript need to be shortened.

**Author’s response:**

Thank you for this valuable comment. We carefully revised the manuscript to improve conciseness while preserving the essential methodological details, key findings, and scientific interpretation. The introduction was shortened by reducing detailed pathophysiological explanations and consolidating the overview of PMS management practices. The Methods section was condensed by simplifying the descriptions of validated assessment tools, questionnaire development, data collection, data analysis, and operational definitions. In the Results section, repetitive narrative descriptions of tabulated findings were reduced, and detailed information on management practices was consolidated to emphasize the most clinically relevant findings. The Discussion was also substantially shortened by reducing repetition, streamlining comparisons with previous studies, and focusing on the independent predictors and principal management findings. These revisions improved the overall clarity, flow, and readability of the manuscript.

2- Some tables could be merged together into one table. For example, the score of a certain scale can be used instead of showing each factor by itself (when applicable).

**Author’s response:**

Thank you for this valuable comment. We reviewed the tables to reduce unnecessary detail and improve the clarity of the main manuscript. Item-level results for the Bergen Social Media Addiction Scale and the Perceived Stress Scale-4 were moved to the Supporting Information, while their total scores were retained in the main text. In addition, detailed PMDD-specific regression analyses and the perceived effectiveness of lifestyle modifications were transferred to supplementary tables. These revisions reduced table length and repetition while preserving the key analyses and transparency of the study findings.

3- Better to mention the name of the university, as this would give a clearer understanding for readers.

**Author’s response:**

Thank you for this helpful comment. We agree that identifying the study setting provides clearer context for readers. Accordingly, the name of the university has been added to the Methods section under “Study design and setting” and is now specified as Beirut Arab University, Lebanon. As follows:

**Study design and setting**

“An observational cross-sectional study using convenience sampling was conducted among female students at Beirut Arab University, Lebanon, from April 20 to May 8, 2026. Beirut Arab University has four campuses distributed across Lebanon, and data were collected from students at all four campuses. The university enrolls students from diverse Lebanese regions and socioeconomic and cultural backgrounds.”

**Reviewers’ comments:**

**Reviewer #1**

1- The manuscript states that the study was conducted among female students at “a university in Lebanon” using convenience sampling, but the participating university is not clearly identified in the Methods section. Please specify the name of the university or clarify whether the data were collected from only one institution. If only one university was included, the authors should justify why other universities were not included and acknowledge that this limits the generalizability of the findings to all female university students in Lebanon.

**Author’s response:**

Thank you for this important comment. We have clarified in the Methods section that the study was conducted at Beirut Arab University, Lebanon. Although the study involved a single university, Beirut Arab University has four campuses distributed across Lebanon, and data were collected from female students at all four campuses. This approach enabled the inclusion of students from diverse geographical regions and socioeconomic and cultural backgrounds. Nevertheless, we acknowledge that the use of convenience sampling within one university may limit the generalizability of the findings to all female university students in Lebanon. This limitation has been added to the manuscript and the study design and setting has been adjusted as follows:

**Study design and setting**

“An observational cross-sectional study using convenience sampling was conducted among female students at Beirut Arab University, Lebanon, from April 20 to May 8, 2026. Beirut Arab University has four campuses distributed across Lebanon, and data were collected from students at all four campuses. The university enrolls students from diverse Lebanese regions and socioeconomic and cultural backgrounds.”

**Study limitations**

“The study was conducted among female students at a single university in Lebanon. Although data were collected across all four campuses of Beirut Arab University and included students from diverse geographical regions and socioeconomic backgrounds, the use of convenience sampling within one institution may limit the generalizability of the findings to all female university students in Lebanon.”

2- The inclusion criteria currently mention women of reproductive age, 18–45 years, but they do not explicitly state that participants had to be university students. Since the target population is female university students, this should be added to the inclusion criteria.

**Author’s response:**

Thank you for this important comment. We have revised the inclusion criteria to explicitly state that eligible participants were female university students aged 18–45 years who were enrolled at Beirut Arab University during the study period, as follows:

**Inclusion and exclusion criteria**

“The inclusion criteria were female students enrolled at Beirut Arab University, aged 18–45 years.”

3- The exclusion criteria include gynecological conditions, thyroid disorders, diagnosed mental health condition etc…. However, if they were self-reported, this should be acknowledged in the Limitations section as a source of recall bias and misclassification bias.

**Author’s response:**

Thank you for this important comment. The exclusion criteria, including gynecological conditions, thyroid disorders, diagnosed mental health conditions, pregnancy or breastfeeding, and hormonal contraceptive use, were based on self-reported information collected through the questionnaire. We have clarified this in the Methods section and added the potential for recall and misclassification bias related to self-reported exclusion criteria to the Limitations section, as follows:

**Inclusion and exclusion criteria**

“Participants were excluded if they reported gynecological conditions such as endometriosis, uterine fibroids, uterine polyps, polycystic ovary syndrome (PCOS), chronic pelvic inflammatory disease, or amenorrhea. Additionally, females reporting medical conditions, including thyroid disorders or diagnosed mental health conditions (e.g., anxiety or depression), as well as those currently using hormonal medications for contraception, were excluded. Pregnant or breastfeeding women were also excluded.”

**Study limitations**

“In addition, the cross-sectional design and reliance on retrospective self-reporting may have increased the possibility of recall bias. Self-reported medical and gynecological conditions used to determine eligibility may also have resulted in misclassification of some participants.”

4- The authors should state the language in which the questionnaire was distributed. If the questionnaire was administered in Arabic, is the Arabic validated versions of the PSST, BSMAS, and PSS-4 were used, and whether these versions were validated in Lebanon or in Arabic-speaking populations. If translation was performed by the research team, the translation/back-translation process should be described.

**Author’s response:**

Thank you for this important comment. We have clarified in the Methods section that the questionnaire was administered in English. Therefore, Arabic versions or translation/back-translation procedures were not applicable. The PSST, BSMAS, and PSS-4 were administered in their original English versions, as the study population comprised university students able to complete the questionnaire in English. The questionnaire development and structure section was adjusted as follows:

**Questionnaire development and structure**

“A self-administered questionnaire was developed by the research team following a review of relevant literature [5,6,8,35]. It consisted of 39 closed-ended questions covering sociodemographic characteristics, menstrual history, premenstrual symptoms, lifestyle and psychosocial factors, and PMS management practices. The questionnaire was administered in English, and the PSST, BSMAS, and PSS-4 were used in their original English versions. The full questionnaire is provided in **S1 Appendix**.”

5- The manuscript reports that 1,345 students were approached and 1,062 were included after excluding 283 participants. However, it should clarify whether any students refused participation, submitted incomplete forms, or were excluded for missing data.

**Author’s response:**

Thank you for this important comment. We have corrected the wording to clarify that 1,345 students completed the electronic questionnaire, rather than being approached for participation. Of these respondents, 283 were excluded based on the predefined eligibility criteria, resulting in 1,062 participants in the final analysis. Participation refusal was therefore not applicable to the analyzed sample. In addition, the electronic questionnaire was designed with mandatory responses for all study variables; consequently, incomplete questionnaires and missing data were not possible at submission. We adjusted the participant enrollment and study population section as follows:

**Participant enrollment and study population**

“A total of 1,345 female participants completed the electronic questionnaire. Of these, 283 participants were excluded for not meeting the inclusion criteria, including participants with PCOS (n = 152), history of psychiatric illness (n = 37), hypothyroidism or hyperthyroidism (n = 36), pregnancy or breastfeeding (n = 30), amenorrhea (n = 13), endometriosis (n = 9), uterine fibroids (n = 5), and chronic pelvic inflammation (n = 1). Therefore, 1,062 participants were included in the final analysis. The electronic questionnaire required completion of all mandatory items before submission; thus, no questionnaires were excluded because of incomplete responses or missing data. Further details regarding participant enrollment and analysis are presented in Fig 1.”

6- The manuscript states that ethical approval was obtained from the Institutional Review Board at Beirut Arab University, but that no approval code was issued. According to PLOS ONE guidance, authors should submit documentation from the ethics committee confirming approval of human-subjects research, with any participant-identifying information redacted. ([PLOS](https://journals.plos.org/plosone/s/human-subjects-research?utm_source=chatgpt.com)) Therefore, the authors should provide a copy of the IRB approval letter or official documentation confirming approval.

**Author’s response:**

Thank you for this important comment. We confirm that ethical approval for this study was obtained from the Institutional Review Board of Beirut Arab University on April 17, 2026. Although no approval reference number was issued, the Institutional Review Board provided official written confirmation that the study was reviewed and approved in accordance with institutional regulations and that data collection was authorized to proceed. A copy of the official IRB approval documentation was submitted with the initial manuscript submission. We will be pleased to re-upload this documentation.

7- The Results section is very detailed and reads more like a thesis than a research manuscript. The authors should shorten the Results section and focus only on the findings directly related to the main research objectives: PMS/PMDD prevalence, associated factors, and management practices.

**Author’s response:**

Thank you for this valuable comment. We substantially revised the Results section to focus on the primary study objectives: the prevalence of moderate-to-severe PMS and PMDD, associated factors, and management practices. Detailed item-level findings for the Bergen Social Media Addiction Scale and Perceived Stress Scale-4 were moved to the Supporting Information, while their overall scores were retained in the main text. In addition, detailed PMDD-specific analyses and the perceived effectiveness of lifestyle modifications were transferred to supplementary tables. Repetitive narrative descriptions of tabulated findings were also reduced.

8- The percentages of students from each faculty or academic field are not equally distributed. This may affect the representativeness of the sample, especially because health-related students may have better awareness of PMS and management options compared with non-medical students. This should be acknowledged in the Limitations section.

**Author’s response:**

Thank you for this important comment. We acknowledge that the distribution of participants across faculties and academic fields was not uniform. Although participants were classified as medical/health-related or non-medical students and academic specialization was not significantly associated with moderate-to-severe PMS/PMDD in the present analysis, health-related students may have greater awareness of PMS symptoms and management options. We have added this as a limitation of the study, as follows:

**Study limitations**

“In addition, the unequal distribution of participants across faculties and academic fields may have affected sample representativeness, as health-related students may have greater awareness of PMS symptoms and management options than non-medical students.”

9- In the academic level variable, the manuscript includes both “Freshman” and “First year.” The difference between these two categories may not be clear to international readers. The authors should define these categories.

**Author’s response:**

Thank you for this helpful comment. We have clarified the academic-level categories by adding an explanatory note to Table 1, as follows:

**Table 1.**

““Freshman” refers to students enrolled in the university’s preparatory foundation year before entry into a degree program, whereas “First year” refers to the first year of an undergraduate degree program.”

10- Several tables are crowded and difficult to read. The table format should be improved. The manuscript would benefit from a more organized presentation of the essential findings.

**Author’s response:**

Thank you for this valuable comment. We carefully revised the presentation and formatting of the tables to improve readability and emphasize the essential findings. Detailed item-level results for the Bergen Social Media Addiction Scale and the Perceived Stress Scale-4 were moved to the Supporting Information, while only the total scores were retained in the main text. In addition, detailed PMDD-specific analyses and perceived effectiveness data were transferred to supplementary tables.

11- The manuscript should present only the essential tables that directly address the research questions and hypotheses. Detailed item-by-item tables, such as Table 3 on premenstrual symptoms, Table 4 on interference with life, and Table 6 on BSMAS items, may be included as supplementary materials. The main manuscript can summarize the key findings from these tables in the text.

**Author’s response:**

Thank you for this valuable comment. We reviewed the tables to ensure that the main manuscript focuses on findings directly relevant to the study objectives. The detailed item-level results for the Bergen Social Media Addiction Scale were moved to the Supporting Information, and only the overall BSMAS score was retained in the main text. Similarly, the item-level Perceived Stress Scale-4 results were transferred to the Supporting Information, with only the total PSS-4 score reported in the manuscript.

Tables 3 and 4 were retained in the main manuscript because they present the frequency and severity of premenstrual symptoms and their functional interference, which are central to the classification of PMS and PMDD using the PSST and directly address the primary objective of estimating the burden of clinically significant premenstrual symptoms.

12- Both the Results and Discussion sections should be shortened to improve readability. The Discussion should focus more on interpreting the main findings, comparing only the most relevant studies, explaining possible mechanisms, and highlighting implications. Repetition of descriptive findings should be reduced.

**Author’s response:**

Thank you for this valuable comment. We revised both the Results and Discussion sections to improve readability and reduce repetition. In the Results section, detailed item-level findings for the BSMAS and PSS-4, as well as selected detailed analyses, were transferred to the Supporting Information, while the main manuscript now emphasizes PMS/PMDD prevalence, associated factors, and management practices.

The Discussion was moderately shortened by removing repetitive descriptive statements, overlapping explanations, and overly detailed examples while retaining the interpretation of the main findings. Comparisons with previous studies were streamlined to prioritize the most relevant regional and international evidence. The revised Discussion continues to address possible mechanisms and implications for university-based awareness, prevention, and management strategies, while reducing repetition of findings already presented in the Results section.

13- Section 3.10 on war-related consequences appears descriptive and not central to the main research questions unless it is included in the main analytical model or hypothesis. If war exposure was not significantly associated with PMS/PMDD or is not a main focus of the study, this section should be removed from the main Results or moved to supplementary material.

**Author’s response:**

Thank you for this helpful comment. We agree that the descriptive section on war-related consequences was not central to the primary study objectives. Accordingly, this subsection has been removed from the main Results section.

14- The manuscript includes several analyses related to PMS and PMDD. To avoid an excessive number of tables, the authors should consider presenting one compact table comparing the main groups: no/mild PMS, moderate-to-severe PMS, and PMDD.

**Author’s response:**

Thank you for this helpful suggestion. We considered presenting a single compact table comparing no/mild PMS, moderate-to-severe PMS, and PMDD. However, to avoid duplication and an excessive number of tables, we retained the main analysis comparing no/mild PMS with moderate-to-severe PMS/PMDD, which directly addresses the primary study objective. Detailed PMDD-specific univariate and multivariable analyses were transferred to the Supporting Information and are now summarized briefly in the main text, as follows:

**Predictors associated with PMDD**

“Univariate associations with PMDD are presented in **S3 Table**. In the multivariable analysis, heavy menstrual bleeding, current smoking, regular exercise, higher BSMAS total score, and higher PSS-4 total score were independently associated with PMDD (**S4 Table**).”

15- Since the study used a self-administered screening tool rather than prospective daily symptom ratings or clinical diagnosis, the authors should avoid saying that participants “met the criteria for PMDD” or were “diagnosed with PMS/PMDD.” More cautious wording such as “screened positive for moderate-to-severe PMS” or “had symptoms consistent with PMDD based on the PSST” would be more appropriate. (For example, in the abstract, replace “met the criteria for PMDD” with “screened positive for symptoms consistent with PMDD.)

**Author’s response:**

Thank you for this important comment. We agree that the PSST is a screening instrument and does not establish a clinical diagnosis of PMS or PMDD. Accordingly, we revised the manuscript throughout to use cautious terminology. Participants are now described as having “moderate-to-severe PMS based on the PSST” or as having “symptoms consistent with PMDD based on the PSST”.

16- The limitation section already acknowledges that the single-university setting may reduce generalizability.

**Author’s response:**

Thank you for noting this. We have retained and further clarified this limitation in the revised manuscript. Specifically, we state that, although data were collected across all four campuses of Beirut Arab University and included students from diverse geographical regions and socioeconomic backgrounds, the use of convenience sampling within a single institution may limit the generalizability of the findings to all female university students in Lebanon.

**Reviewer #2**

1- The aims could be presented in a straight forward way at the end of the introduction.

**Author’s response:**

Thank you for this helpful comment. We revised the final paragraph of the Introduction to present the study aims more directly and clearly**,** as follows:

**Introduction**

“Therefore, this study aimed to estimate the prevalence of moderate-to-severe PMS and symptoms consistent with PMDD among female university students in Lebanon, identify associated factors, and describe management practices and their perceived effectiveness.”

2- Some sections would benefit from minimizing, such as the introduction and discussion.

**Author’s response:**

Thank you for this helpful comment. We revised the Introduction and Discussion to improve conciseness while preserving the essential rationale, interpretation, and clinical relevance of the study.

3- Some tables can be merged to lower the number of tables, as some tables are of 1 row only.

**Author’s response:**

Thank you for this helpful comment. We reviewed the tables to reduce their number and avoid standalone tables presenting limited information. Detailed item-level scale results were moved to the Supporting Information, and the perceived effectiveness of lifestyle modifications, which was previously presented as a single-outcome table, was also transferred to the supplementary material. The main manuscript now retains only tables that directly support the primary objectives, including participant characteristics, menstrual characteristics, PMS symptom burden and functional interference, lifestyle patterns, and the main univariate and multivariable analyses.

4- Section 2.3: While the second section included menstrual history characteristics such as age at menarche, menstrual cycle duration, length of menstrual bleeding, and heaviness of blood flow. Language mistake

**Author’s response:**

Thank you for identifying this language issue. We revised the entire section to improve clarity, grammar, and conciseness. The revised section now describes the questionnaire structure and assessment tools in a clear and organized manner, including the menstrual-history component, without using the previously unclear wording.

5- The first paragraph of the discussion should summarize the findings instead of delving in the comparison.

**Author’s response:**

Thank you for this helpful comment. We revised the opening of the Discussion to provide a concise summary of the main study findings before comparing them with previous literature. The revised first paragraph now highlights the prevalence of moderate-to-severe PMS and symptoms consistent with PMDD, the most frequently reported symptoms, the impact on academic functioning, the main associated factors, and the key management findings, as follows:

**Discussion**

“This study has demonstrated the presence of a substantial burden of premenstrual symptoms among female university students in Lebanon. Nearly half of the participants screened positive for moderate-to-severe PMS, and 16.5% had symptoms consistent with PMDD based on the PSST. The most frequently reported physical manifestations were fatigue, depressed mood, and food cravings, and more than one-third of participants reported PMS-related academic absenteeism. Heavy menstrual bleeding, current smoking, meal skipping, frequent fast-food consumption, higher social media addiction scores, and higher perceived stress scores were independently associated with moderate-to-severe PMS/PMDD. Although lifestyle modifications were used by almost half of participants with clinically significant symptoms, pharmacological management was reported by 39.0%, mainly through analgesic use, and professional consultation was limited.**”**
