# Supplementary material for: Prevalence, risk factors, and management practices of premenstrual syndrome among female university students in Lebanon: An observational cross-sectional study
Source: PLoS One. 2026 Jul 27;21(7):e0354807. doi: 10.1371/journal.pone.0354807 (PMC13405303; doi:10.1371/journal.pone.0354807)
Supplement: Attachment — Submitted filename: Comments - PMS.docx [file pone.0354807.s005.docx]

The manuscript addresses an important and underexplored topic in Lebanon: PMS prevalence, associated factors, and management practices among female university students. The study has a large sample size and uses recognized tools such as the PSST, BSMAS, and PSS-4 to assess premenstrual symptoms, social media addiction, and perceived stress. However, several methodological and reporting issues should be addressed before the manuscript can be considered for publication.

1. The manuscript states that the study was conducted among female students at “a university in Lebanon” using convenience sampling, but the participating university is not clearly identified in the Methods section. Please specify the name of the university or clarify whether the data were collected from only one institution. If only one university was included, the authors should justify why other universities were not included and acknowledge that this limits the generalizability of the findings to all female university students in Lebanon.
2. The inclusion criteria currently mention women of reproductive age, 18–45 years, but they do not explicitly state that participants had to be university students. Since the target population is female university students, this should be added to the inclusion criteria.
3. The exclusion criteria include gynecological conditions, thyroid disorders, diagnosed mental health condition etc…. However, if they were self-reported, this should be acknowledged in the Limitations section as a source of recall bias and misclassification bias.
4. The authors should state the language in which the questionnaire was distributed. If the questionnaire was administered in Arabic, is the Arabic validated versions of the PSST, BSMAS, and PSS-4 were used, and whether these versions were validated in Lebanon or in Arabic-speaking populations. If translation was performed by the research team, the translation/back-translation process should be described.
5. The manuscript reports that 1,345 students were approached and 1,062 were included after excluding 283 participants. However, it should clarify whether any students refused participation, submitted incomplete forms, or were excluded for missing data.
6. The manuscript states that ethical approval was obtained from the Institutional Review Board at Beirut Arab University, but that no approval code was issued. According to PLOS ONE guidance, authors should submit documentation from the ethics committee confirming approval of human-subjects research, with any participant-identifying information redacted. ([PLOS](https://journals.plos.org/plosone/s/human-subjects-research?utm_source=chatgpt.com)) Therefore, the authors should provide a copy of the IRB approval letter or official documentation confirming approval.
7. The Results section is very detailed and reads more like a thesis than a research manuscript. The authors should shorten the Results section and focus only on the findings directly related to the main research objectives: PMS/PMDD prevalence, associated factors, and management practices.
8. The percentages of students from each faculty or academic field are not equally distributed. This may affect the representativeness of the sample, especially because health-related students may have better awareness of PMS and management options compared with non-medical students. This should be acknowledged in the Limitations section.
9. In the academic level variable, the manuscript includes both “Freshman” and “First year.” The difference between these two categories may not be clear to international readers. The authors should define these categories.
10. Several tables are crowded and difficult to read. The table format should be improved. The manuscript would benefit from a more organized presentation of the essential findings.
11. The manuscript should present only the essential tables that directly address the research questions and hypotheses. Detailed item-by-item tables, such as Table 3 on premenstrual symptoms, Table 4 on interference with life, and Table 6 on BSMAS items, may be included as supplementary materials. The main manuscript can summarize the key findings from these tables in the text.
12. Both the Results and Discussion sections should be shortened to improve readability. The Discussion should focus more on interpreting the main findings, comparing only the most relevant studies, explaining possible mechanisms, and highlighting implications. Repetition of descriptive findings should be reduced.
13. Section 3.10 on war-related consequences appears descriptive and not central to the main research questions unless it is included in the main analytical model or hypothesis. If war exposure was not significantly associated with PMS/PMDD or is not a main focus of the study, this section should be removed from the main Results or moved to supplementary material.
14. The manuscript includes several analyses related to PMS and PMDD. To avoid an excessive number of tables, the authors should consider presenting one compact table comparing the main groups: no/mild PMS, moderate-to-severe PMS, and PMDD.
15. Since the study used a self-administered screening tool rather than prospective daily symptom ratings or clinical diagnosis, the authors should avoid saying that participants “met the criteria for PMDD” or were “diagnosed with PMS/PMDD.” More cautious wording such as “screened positive for moderate-to-severe PMS” or “had symptoms consistent with PMDD based on the PSST” would be more appropriate. (For example, in the abstract, replace “met the criteria for PMDD” with “screened positive for symptoms consistent with PMDD.)
16. The limitation section already acknowledges that the single-university setting may reduce generalizability.

Regards,
