# Supplementary material for: Prevalence, risk factors, and management practices of premenstrual syndrome among female university students in Lebanon: An observational cross-sectional study
Source: PLoS One. 2026 Jul 27;21(7):e0354807. doi: 10.1371/journal.pone.0354807 (PMC13405303; doi:10.1371/journal.pone.0354807)
Supplement: Attachment — Submitted filename: Responses to Reviewers Comments - Second Round PO.docx [file pone.0354807.s008.docx]

**Dear Prof. Jad El Masri**
Academic Editor
PLOS One

PONE-D-26-25846R1

Editor’s comments

I congratulate the authors as the manuscript has clearly improved. Below are some minor comments, before the manuscript can move forward. Best of luck

I am pleasured to re-review this manuscript. I believe that the authors succeeded in making substantial improvements to their work. I still have some comments/suggestions that can help improve the work, before it can move forward.

Thank you for giving us the opportunity to submit a second revised version of our manuscript entitled “**Prevalence, risk factors, and management practices of premenstrual syndrome among female university students in Lebanon: an observational cross-sectional study”** to PLOS One*.*

We sincerely appreciate the time and effort that you and the reviewers have dedicated to re-evaluating our manuscript. We are especially grateful for your positive assessment, acknowledging the substantial improvements made to our work. We highly value the constructive comments and suggestions provided, which have further strengthened the quality and clarity of the manuscript.

We have carefully considered all of the remaining comments and suggestions and have incorporated the recommended revisions throughout the manuscript. All modifications have been highlighted in the revised version for ease of review.‎

Please see below, in **blue**, for **point-by-point responses** to the comments and ‎concerns.

Please find below my comments:

1. The introduction/discussion need to be shortened.

**Authors’ response:** Thank you for this constructive suggestion and for the positive evaluation of our manuscript. In response, we have carefully revised the manuscript to improve its conciseness and readability. Specifically, the **Introduction** has been streamlined by removing redundant background information and retaining only the most relevant literature to better establish the study rationale. In addition, the **Discussion** has been substantially shortened by eliminating repetitive statements, reducing unnecessary descriptive comparisons, and focusing on the interpretation and implications of the principal findings.

2. The limitations section could benefit from mentioning that BAU does not represent the Lebanese population, especially since it is a private university. Usually, the financial state is a major stress factor, so the chosen population may differ from other populations, such as public university students. Despite not being a major point, the paper would benefit from adding this explanation into the limitations.

**Authors’ response:** We sincerely thank you for this valuable suggestion. We agree that the study population may not fully represent all female university students in Lebanon, particularly those enrolled in public universities or institutions with different socioeconomic profiles. Accordingly, we have revised the limitations section to acknowledge that the study was conducted at a single private university, which may limit the generalizability of the findings. We also noted that socioeconomic differences, including financial stressors, may influence the prevalence and experience of premenstrual syndrome and should be considered when interpreting the results. We have further recommended that future studies include students from both public and private universities across different regions of Lebanon to enhance the representativeness and generalizability of the findings.

Revised text in the manuscript, under the study limitations section:

“The study was conducted among female students at a single university in Lebanon. Although data were collected across all four campuses of Beirut Arab University and included students from diverse geographical regions, the use of convenience sampling within one institution may limit the generalizability of the findings to all female university students in Lebanon. Furthermore, because Beirut Arab University is a private institution, the study population may not fully represent students enrolled in public universities or other higher education institutions in Lebanon. Differences in socioeconomic characteristics and financial stressors between students attending public and private universities may influence the prevalence and severity of PMS and PMDD and should therefore be considered when interpreting the findings.”

“Future multicenter studies involving students from diverse educational institutions, including both public and private universities, and different geographic regions across Lebanon are warranted to provide more representative national estimates and enhance the generalizability of the findings.”

3. Similar to figure 2, I suggest making similar representation for all the scales used. This is needed to give the readers general idea about the levels in the sample targeted, before delving into comparisons.

**Authors’ Response:** We sincerely thank you for this valuable suggestion. In response to your and reviewer # 2 recommendation, we have added graphical representations of the distributions of participants' responses for both the Bergen Social Media Addiction Scale (BSMAS) and the Perceived Stress Scale-4 (PSS-4) by including **Figures 3** and **4**, respectively. These figures provide readers with a clear overview of the distribution of responses across the study sample before presenting the comparative and regression analyses. The corresponding descriptions and citations have also been incorporated into the Results section.

4. Tables showing frequency of answers on each item of the scale could be moved to supplementary, and those having univariate and multivariate could be in the main manuscript instead of the supplementary, as these give more information and offer greater value to readers.
   Looking forward to reading the revised version
   Best of luck

**Authors’ response:** We sincerely thank you for this valuable suggestion. In response, we have reorganized the presentation of the tables to improve the readability and overall flow of the manuscript. Specifically, the detailed tables presenting the frequency distributions of responses to the individual questionnaire items (**PSST; originally Tables 3 and 4**) have been moved to the Supplementary Material, while the univariate and multivariable regression analysis tables related to the predictors of PMDD have been moved to the main manuscript, as these represent the primary analytical findings of the study and provide greater value to readers. Accordingly, the table numbering and all corresponding in-text citations have been revised throughout the manuscript to reflect these changes. We appreciate this helpful recommendation, which has enhanced the clarity and organization of the manuscript.

[Note: HTML markup is below. Please do not edit.]

Reviewers' comments:

Reviewer's Responses to Questions

**Comments to the Author**

1. If the authors have adequately addressed your comments raised in a previous round of review and you feel that this manuscript is now acceptable for publication, you may indicate that here to bypass the “Comments to the Author” section, enter your conflict of interest statement in the “Confidential to Editor” section, and submit your "Accept" recommendation.

Reviewer #2: All comments have been addressed

2. Is the manuscript technically sound, and do the data support the conclusions?

The manuscript must describe a technically sound piece of scientific research with data that supports the conclusions. Experiments must have been conducted rigorously, with appropriate controls, replication, and sample sizes. The conclusions must be drawn appropriately based on the data presented.

Reviewer #2: Yes

3. Has the statistical analysis been performed appropriately and rigorously?

Reviewer #2: Yes

4. Have the authors made all data underlying the findings in their manuscript fully available?

The [PLOS Data policy](https://eur01.safelinks.protection.outlook.com/?url=http%3A%2F%2Ftrack.editorialmanager.com%2FCL0%2Fhttp%3A%252F%252Fwww.plosone.org%252Fstatic%252Fpolicies.action%2523sharing%2F1%2F010f019f125cd071-6946ed8a-b957-48b9-906b-0e6811180970-000000%2Fvecov66F2VMfRWoLFCbkrjiEfozmoHjqr3eKe6xU_oQ%3D258&data=05%7C02%7Cr.itani%40bau.edu.lb%7C89d9252b432f439bc3ba08ded5b34204%7Cae73320887834b9394a5c5f29fb71fa9%7C0%7C0%7C639183163123636789%7CUnknown%7CTWFpbGZsb3d8eyJFbXB0eU1hcGkiOnRydWUsIlYiOiIwLjAuMDAwMCIsIlAiOiJXaW4zMiIsIkFOIjoiTWFpbCIsIldUIjoyfQ%3D%3D%7C0%7C%7C%7C&sdata=Abl2S1%2FqyQi58oSBDqrCdu4CCyixTOSVArifsmfqrPk%3D&reserved=0) requires authors to make all data underlying the findings described in their manuscript fully available without restriction, with rare exception (please refer to the Data Availability Statement in the manuscript PDF file). The data should be provided as part of the manuscript or its supporting information, or deposited to a public repository. For example, in addition to summary statistics, the data points behind means, medians and variance measures should be available. If there are restrictions on publicly sharing data—e.g. participant privacy or use of data from a third party—those must be specified.

Reviewer #2: No

**Authors' Response:** We sincerely thank you for highlighting this important point. In response, we have now made the underlying data supporting the findings of this study fully available by providing the complete dataset as Supporting Information in accordance with the PLOS ONE Data Availability Policy. The Data Availability Statement has also been revised accordingly to reflect this update.

As follow: “**Availability of data and materials**: All data underlying the findings of this study are fully available without restriction. The complete de-identified dataset has been provided as **S1 Dataset** in the Supporting Information.”

5. Is the manuscript presented in an intelligible fashion and written in standard English?

PLOS ONE does not copyedit accepted manuscripts, so the language in submitted articles must be clear, correct, and unambiguous. Any typographical or grammatical errors should be corrected at revision, so please note any specific errors here.

Reviewer #2: Yes

6. Review Comments to the Author

Please use the space provided to explain your answers to the questions above. You may also include additional comments for the author, including concerns about dual publication, research ethics, or publication ethics. (Please upload your review as an attachment if it exceeds 20,000 characters)

Reviewer #2: I congratulate authors on the progress made. I believe that making some minor changes would make the paper good to go.

**Authors' Response:** We sincerely thank you for the careful re-evaluation of our manuscript and for the encouraging and positive feedback. We greatly appreciate the recognition of the progress made in improving the manuscript. We have carefully considered all of the remaining comments and suggestions and have revised the manuscript accordingly. Our detailed point-by-point responses are provided below.

1. The keywords could benefit from adding "University students" and removing abbreviated words as "SDG"

**Authors' Response:** We sincerely thank you for this helpful suggestion. In response, we have revised the keywords by removing the abbreviation **“SDG”** and by adding **“University Students”**.

2. I agree that some tables should be removed to the supplementary part, but those having univariate and multivariate are worth keeping in my opinion. Those specifiying individual questions in the scale could be replaced with a figure showing the population's level, and these tables could be moved to the supplementary part.

**Authors' Response:** Thank you for this valuable suggestion. In response, we have reorganized the presentation of the results to improve the readability and overall flow of the manuscript. Specifically, the detailed tables presenting the frequency distributions of responses to the individual PSST items (formerly Tables 3 and 4) have been moved to the Supplementary Material. In addition, we have added **Figures 3** and **4**, which graphically present the distributions of participants' responses to the Bergen Social Media Addiction Scale (BSMAS) and the Perceived Stress Scale-4 (PSS-4), respectively, providing readers with an overall overview of the response distributions before the comparative analyses. Furthermore, the univariate and multivariable regression analysis tables have been retained in the main manuscript, as recommended. The table numbering and corresponding in-text citations have also been updated throughout the manuscript to reflect these changes.

3. The introduction could benefit from shortening, and the discussion needs to be shortened a lot (11 pages in its current form).
   I believe that the manuscript is interesting and offers an addition to the literature after making these minor changes.
   Good luck

**Authors' Response:** Thank you for this constructive suggestion and for the positive evaluation of our manuscript. In response, we have carefully revised the manuscript to improve its conciseness and readability. Specifically, the **Introduction** has been streamlined by removing redundant background information and retaining only the most relevant literature to better establish the study rationale. In addition, the **Discussion** has been substantially shortened by eliminating repetitive statements, reducing unnecessary descriptive comparisons, and focusing on the interpretation and implications of the principal findings.

7. PLOS authors have the option to publish the peer review history of their article ([what does this mean?](https://eur01.safelinks.protection.outlook.com/?url=https%3A%2F%2Ftrack.editorialmanager.com%2FCL0%2Fhttps%3A%252F%252Fjournals.plos.org%252Fplosone%252Fs%252Feditorial-and-peer-review-process%2523loc-peer-review-history%2F1%2F010f019f125cd071-6946ed8a-b957-48b9-906b-0e6811180970-000000%2Fn4UwyM2hYGZ1qhQ-OJBhOuYmrys9TNLxpUr3I4sUb7Q%3D258&data=05%7C02%7Cr.itani%40bau.edu.lb%7C89d9252b432f439bc3ba08ded5b34204%7Cae73320887834b9394a5c5f29fb71fa9%7C0%7C0%7C639183163123667500%7CUnknown%7CTWFpbGZsb3d8eyJFbXB0eU1hcGkiOnRydWUsIlYiOiIwLjAuMDAwMCIsIlAiOiJXaW4zMiIsIkFOIjoiTWFpbCIsIldUIjoyfQ%3D%3D%7C0%7C%7C%7C&sdata=bAwg4INjwqIEdzOIopbzRyT1G8bKnFwPacuguXMwAjo%3D&reserved=0)). If published, this will include your full peer review and any attached files.


If you choose “no”, your identity will remain anonymous but your review may still be made public.


**Do you want your identity to be public for this peer review?** For information about this choice, including consent withdrawal, please see our [Privacy Policy](https://eur01.safelinks.protection.outlook.com/?url=https%3A%2F%2Ftrack.editorialmanager.com%2FCL0%2Fhttps%3A%252F%252Fwww.plos.org%252Fprivacy-policy%2F1%2F010f019f125cd071-6946ed8a-b957-48b9-906b-0e6811180970-000000%2FSA1enoIjFQu37A37LXtrKO5VMeOyaTLi9FKDxpunYJo%3D258&data=05%7C02%7Cr.itani%40bau.edu.lb%7C89d9252b432f439bc3ba08ded5b34204%7Cae73320887834b9394a5c5f29fb71fa9%7C0%7C0%7C639183163123696622%7CUnknown%7CTWFpbGZsb3d8eyJFbXB0eU1hcGkiOnRydWUsIlYiOiIwLjAuMDAwMCIsIlAiOiJXaW4zMiIsIkFOIjoiTWFpbCIsIldUIjoyfQ%3D%3D%7C0%7C%7C%7C&sdata=elOejSJsS%2F5%2BoErwaX8bbWD3z0pv0Aqup2NmRz4LZfQ%3D&reserved=0).

Reviewer #2: No

[NOTE: If reviewer comments were submitted as an attachment file, they will be attached to this email and accessible via the submission site. Please log into your account, locate the manuscript record, and check for the action link "View Attachments". If this link does not appear, there are no attachment files.]

To ensure your figures meet our technical requirements, please review our figure guidelines: <https://journals.plos.org/plosone/s/figures>

You may also use PLOS’s free figure tool, NAAS, to help you prepare publication quality figures: <https://journals.plos.org/plosone/s/figures#loc-tools-for-figure-preparation>.

NAAS will assess whether your figures meet our technical requirements by comparing each figure against our figure specifications.
