# Supplementary material for: Prevalence, risk factors, and management practices of premenstrual syndrome among female university students in Lebanon: An observational cross-sectional study
Source: PLoS One. 2026 Jul 27;21(7):e0354807. doi: 10.1371/journal.pone.0354807 (PMC13405303; doi:10.1371/journal.pone.0354807)
Supplement: Peer review history

# Peer review: Decision Letter 0

**DOI:** 10.1371/journal.pone.0354807.r001

**Authors:** Jad El Masri

21 Jun 2026

PONE-D-26-25846

Prevalence, Risk Factors, and Management Practices of Premenstrual Syndrome among Female University Students in Lebanon: An Observational Cross-Sectional Study

PLOS One

Dear Dr. Itani,

Thank you for submitting your manuscript to PLOS ONE. After careful consideration, we feel that it has merit but does not fully meet PLOS ONE’s publication criteria as it currently stands. Therefore, we invite you to submit a revised version of the manuscript that addresses the points raised during the review process.

The manuscript would benefit from making some changes, before it can be accepted. Please make sure to shorten most sections and limit the number of tables (when applicable).

Please submit your revised manuscript by Aug 20 2026 11:59PM. If you will need more time than this to complete your revisions, please reply to this message or contact the journal office at plosone@plos.org. When you're ready to submit your revision, log on to https://www.editorialmanager.com/pone/ and select the 'Submissions Needing Revision' folder to locate your manuscript file.

Please include the following items when submitting your revised manuscript:

- A letter that responds to each point raised by the academic editor and reviewer(s). You should upload this letter as a separate file labeled 'Response to Reviewers'.
- A marked-up copy of your manuscript that highlights changes made to the original version. You should upload this as a separate file labeled 'Revised Manuscript with Track Changes'.
- An unmarked version of your revised paper without tracked changes. You should upload this as a separate file labeled 'Manuscript'.

If you would like to make changes to your financial disclosure, please include your updated statement in your cover letter. Guidelines for resubmitting your figure files are available below the reviewer comments at the end of this letter.

If applicable, we recommend that you deposit your laboratory protocols in protocols.io to enhance the reproducibility of your results. Protocols.io assigns your protocol its own identifier (DOI) so that it can be cited independently in the future. For instructions see: https://journals.plos.org/plosone/s/submission-guidelines#loc-laboratory-protocols. Additionally, PLOS ONE offers an option for publishing peer-reviewed Lab Protocol articles, which describe protocols hosted on protocols.io. Read more information on sharing protocols at https://plos.org/protocols?utm_medium=editorial-email&utm_source=authorletters&utm_campaign=protocols.

As the corresponding author, your ORCID iD is verified in the submission system and will appear in the published article. PLOS supports the use of ORCID, and we encourage all coauthors to register for an ORCID iD and use it as well. Please encourage your coauthors to verify their ORCID iD within the submission system before final acceptance, as unverified ORCID iDs will not appear in the published article. *Only*  the individual author can complete the verification step; PLOS staff *cannot*  verify ORCID iDs on behalf of authors.

We look forward to receiving your revised manuscript.

Kind regards,

Jad El Masri

Academic Editor

PLOS One

Journal Requirements:

When submitting your revision, we need you to address these additional requirements.

1. Please ensure that your manuscript meets PLOS ONE's style requirements, including those for file naming. The PLOS ONE style templates can be found at

https://journals.plos.org/plosone/s/file?id=wjVg/PLOSOne_formatting_sample_main_body.pdf and

https://journals.plos.org/plosone/s/file?id=ba62/PLOSOne_formatting_sample_title_authors_affiliations.pdf

2. In the online submission form you indicate that your data is not available for proprietary reasons and have provided a contact point for accessing this data. Please note that your current contact point is a co-author on this manuscript. According to our Data Policy, the contact point must not be an author on the manuscript and must be an institutional contact, ideally not an individual. Please revise your data statement to a non-author institutional point of contact, such as a data access or ethics committee, and send this to us via return email. Please also include contact information for the third party organization, and please include the full citation of where the data can be found.

3. Your ethics statement should only appear in the Methods section of your manuscript. If your ethics statement is written in any section besides the Methods, please move it to the Methods section and delete it from any other section. Please ensure that your ethics statement is included in your manuscript, as the ethics statement entered into the online submission form will not be published alongside your manuscript.

4. Please include captions for your Supporting Information files at the end of your manuscript, and update any in-text citations to match accordingly. Please see our Supporting Information guidelines for more information: http://journals.plos.org/plosone/s/supporting-information.

5. If the reviewer comments include a recommendation to cite specific previously published works, please review and evaluate these publications to determine whether they are relevant and should be cited. There is no requirement to cite these works unless the editor has indicated otherwise.

Additional Editor Comments:

Authors tackle an important point in the Lebanese population. The manuscript is well-prepared and have the potential to be published following some edits. In addition to the reviewers' comments, below are some points to highlight:

1- Most sections of the manuscript need to be shortened.

2- Some tables could be merged together into one table. For example, the score of a certain scale can be used instead of showing each factor by itself (when applicable).

3- Better to mention the name of the university, as this would give a clearer understanding for readers.

Overall, I believe that this manuscript is important and offers an addition to the literature, especially with the lack of similar studies in the Lebanese population.

[Note: HTML markup is below. Please do not edit.]

Reviewers' comments:

Reviewer's Responses to Questions

**Comments to the Author**

1. Is the manuscript technically sound, and do the data support the conclusions?

The manuscript must describe a technically sound piece of scientific research with data that supports the conclusions. Experiments must have been conducted rigorously, with appropriate controls, replication, and sample sizes. The conclusions must be drawn appropriately based on the data presented.

Reviewer #1: Yes

Reviewer #2: Yes

**********

2. Has the statistical analysis been performed appropriately and rigorously?

Reviewer #1: Yes

Reviewer #2: Yes

**********

3. Have the authors made all data underlying the findings in their manuscript fully available?

The PLOS Data policy requires authors to make all data underlying the findings described in their manuscript fully available without restriction, with rare exception (please refer to the Data Availability Statement in the manuscript PDF file). The data should be provided as part of the manuscript or its supporting information, or deposited to a public repository. For example, in addition to summary statistics, the data points behind means, medians and variance measures should be available. If there are restrictions on publicly sharing data—e.g. participant privacy or use of data from a third party—those must be specified.

Reviewer #1: No

Reviewer #2: No

**********

4. Is the manuscript presented in an intelligible fashion and written in standard English?

PLOS ONE does not copyedit accepted manuscripts, so the language in submitted articles must be clear, correct, and unambiguous. Any typographical or grammatical errors should be corrected at revision, so please note any specific errors here.

Reviewer #1: Yes

Reviewer #2: Yes

**********

5. Review Comments to the Author

Please use the space provided to explain your answers to the questions above. You may also include additional comments for the author, including concerns about dual publication, research ethics, or publication ethics. (Please upload your review as an attachment if it exceeds 20,000 characters)

Reviewer #1: The manuscript addresses an important and underexplored topic in Lebanon: PMS prevalence, associated factors, and management practices among female university students. The study has a large sample size and uses recognized tools such as the PSST, BSMAS, and PSS-4 to assess premenstrual symptoms, social media addiction, and perceived stress. However, several methodological and reporting issues should be addressed before the manuscript can be considered for publication.

1. The manuscript states that the study was conducted among female students at “a university in Lebanon” using convenience sampling, but the participating university is not clearly identified in the Methods section. Please specify the name of the university or clarify whether the data were collected from only one institution. If only one university was included, the authors should justify why other universities were not included and acknowledge that this limits the generalizability of the findings to all female university students in Lebanon.

2. The inclusion criteria currently mention women of reproductive age, 18–45 years, but they do not explicitly state that participants had to be university students. Since the target population is female university students, this should be added to the inclusion criteria.

3. The exclusion criteria include gynecological conditions, thyroid disorders, diagnosed mental health condition etc…. However, if they were self-reported, this should be acknowledged in the Limitations section as a source of recall bias and misclassification bias.

4. The authors should state the language in which the questionnaire was distributed. If the questionnaire was administered in Arabic, is the Arabic validated versions of the PSST, BSMAS, and PSS-4 were used, and whether these versions were validated in Lebanon or in Arabic-speaking populations. If translation was performed by the research team, the translation/back-translation process should be described.

5. The manuscript reports that 1,345 students were approached and 1,062 were included after excluding 283 participants. However, it should clarify whether any students refused participation, submitted incomplete forms, or were excluded for missing data.

6. The manuscript states that ethical approval was obtained from the Institutional Review Board at Beirut Arab University, but that no approval code was issued. According to PLOS ONE guidance, authors should submit documentation from the ethics committee confirming approval of human-subjects research, with any participant-identifying information redacted. (PLOS) Therefore, the authors should provide a copy of the IRB approval letter or official documentation confirming approval.

7. The Results section is very detailed and reads more like a thesis than a research manuscript. The authors should shorten the Results section and focus only on the findings directly related to the main research objectives: PMS/PMDD prevalence, associated factors, and management practices.

8. The percentages of students from each faculty or academic field are not equally distributed. This may affect the representativeness of the sample, especially because health-related students may have better awareness of PMS and management options compared with non-medical students. This should be acknowledged in the Limitations section.

9. In the academic level variable, the manuscript includes both “Freshman” and “First year.” The difference between these two categories may not be clear to international readers. The authors should define these categories.

10. Several tables are crowded and difficult to read. The table format should be improved. The manuscript would benefit from a more organized presentation of the essential findings.

11. The manuscript should present only the essential tables that directly address the research questions and hypotheses. Detailed item-by-item tables, such as Table 3 on premenstrual symptoms, Table 4 on interference with life, and Table 6 on BSMAS items, may be included as supplementary materials. The main manuscript can summarize the key findings from these tables in the text.

12. Both the Results and Discussion sections should be shortened to improve readability. The Discussion should focus more on interpreting the main findings, comparing only the most relevant studies, explaining possible mechanisms, and highlighting implications. Repetition of descriptive findings should be reduced.

13. Section 3.10 on war-related consequences appears descriptive and not central to the main research questions unless it is included in the main analytical model or hypothesis. If war exposure was not significantly associated with PMS/PMDD or is not a main focus of the study, this section should be removed from the main Results or moved to supplementary material.

14. The manuscript includes several analyses related to PMS and PMDD. To avoid an excessive number of tables, the authors should consider presenting one compact table comparing the main groups: no/mild PMS, moderate-to-severe PMS, and PMDD.

15. Since the study used a self-administered screening tool rather than prospective daily symptom ratings or clinical diagnosis, the authors should avoid saying that participants “met the criteria for PMDD” or were “diagnosed with PMS/PMDD.” More cautious wording such as “screened positive for moderate-to-severe PMS” or “had symptoms consistent with PMDD based on the PSST” would be more appropriate. (For example, in the abstract, replace “met the criteria for PMDD” with “screened positive for symptoms consistent with PMDD.)

16. The limitation section already acknowledges that the single-university setting may reduce generalizability.

Regards,

Reviewer #2: This manuscript reports an observational cross-sectional study assessing the prevalence, associated risk factors, and management practices of premenstrual syndrome among female university students in Lebanon. The topic is relevant to women’s health and student well-being, particularly in a setting where local data remain limited, and the manuscript is well-prepared and offers an addition to the literature. Below are some comments that can help improve the work:

1- The aims could be presented in a straight forward way at the end of the introduction.

2- Some sections would benefit from minimizing, such as the introduction and discussion.

3- Some tables can be merged to lower the number of tables, as some tables are of 1 row only.

4- Section 2.3: While the second section included menstrual history characteristics such as age at menarche, menstrual cycle duration, length of menstrual bleeding, and heaviness of blood flow. Language mistake

5- The first paragraph of the discussion should summarize the findings instead of delving in the comparision.

Overall, the manuscript is well prepared and follow the guidelines. Minor revisions could help improve the work.

**********

6. PLOS authors have the option to publish the peer review history of their article (what does this mean?). If published, this will include your full peer review and any attached files.

If you choose “no”, your identity will remain anonymous but your review may still be made public.

**Do you want your identity to be public for this peer review?**  For information about this choice, including consent withdrawal, please see our Privacy Policy.

Reviewer #1: No

Reviewer #2: No

**********

[NOTE: If reviewer comments were submitted as an attachment file, they will be attached to this email and accessible via the submission site. Please log into your account, locate the manuscript record, and check for the action link "View Attachments". If this link does not appear, there are no attachment files.]

To ensure your figures meet our technical requirements, please review our figure guidelines: https://journals.plos.org/plosone/s/figures

You may also use PLOS’s free figure tool, NAAS, to help you prepare publication quality figures: https://journals.plos.org/plosone/s/figures#loc-tools-for-figure-preparation.

NAAS will assess whether your figures meet our technical requirements by comparing each figure against our figure specifications.

Attachment Submitted filename: Comments - PMS.docx

# Author comment: Author response to Decision Letter 1

**DOI:** 10.1371/journal.pone.0354807.r002

24 Jun 2026

Dear Prof. Jad El Masri,

Academic Editor

PLOS ONE

Submission ID #: PONE-D-26-25846

Thank you for giving us the opportunity to submit a revised draft of our manuscript titled “Prevalence, Risk Factors, and Management Practices of Premenstrual Syndrome among Female University Students in Lebanon: An Observational Cross-Sectional Study” to PLOS One. We appreciate the time and effort that you and the reviewers have dedicated to providing valuable feedback on our manuscript. We are grateful to your insightful comments. We have been able to incorporate changes to reflect all of the suggestions you and the reviewers have provided. Those changes are highlighted within the manuscript.

Please see below, in blue, for point-by-point responses to the comments and concerns

Editor’s comments:

1- Please ensure that your manuscript meets PLOS ONE's style requirements, including those for file naming.

Author’s response: Thank you for this important reminder. We carefully reviewed the PLOS ONE style requirements and reformatted the manuscript accordingly. Specifically, we revised the title page, author affiliations, section headings, page numbering, continuous line numbering, spacing, table formatting, figure citations and captions, and reference citation style to align with the journal’s guidelines. All main headings and subheadings were revised to sentence case, and tables were retained as editable tables and placed directly after their first citation in the text. Figure files were prepared for separate upload using the required naming conventions, while their captions were retained in the manuscript. We also reviewed the Supporting Information labels and file names to ensure consistency with PLOS ONE requirements.

2- In the online submission form you indicate that your data is not available for proprietary reasons and have provided a contact point for accessing this data. Please note that your current contact point is a co-author on this manuscript. According to our Data Policy, the contact point must not be an author on the manuscript and must be an institutional contact, ideally not an individual. Please revise your data statement to a non-author institutional point of contact, such as a data access or ethics committee, and send this to us via return email. Please also include contact information for the third party organization, and please include the full citation of where the data can be found.

Author’s response:

Thank you for this important comment. We agree that the Data Availability Statement should identify a non-author institutional contact point and clearly explain the reason for restricted data access. The individual-level dataset contains sensitive information related to participants’ menstrual health, psychosocial characteristics, lifestyle behaviors, and health-related practices. Although no direct identifiers were collected, public sharing of the full dataset may pose a risk of participant re-identification because of the sensitive nature and combination of variables collected. Accordingly, we revised the Data Availability Statement to remove the co-author contact point and to designate the Institutional Review Board of Beirut Arab University as the non-author institutional contact responsible for reviewing requests for access to de-identified data. We also clarified that the data were collected directly by the research team at Beirut Arab University and were not obtained from, owned by, or deposited with a third-party organization. Therefore, no third-party data-source citation, repository DOI, or external dataset citation is applicable. The revised statement is as follows:

Availability of data and materials:

“The individual-level data underlying this study contain sensitive information related to menstrual health, psychosocial characteristics, lifestyle behaviors, and health-related practices. Although direct identifiers were not collected, public sharing of the full dataset may pose a risk of participant re-identification because of the sensitive nature and combination of variables collected. The data were collected directly by the research team at Beirut Arab University and were not obtained from, owned by, or deposited with a third-party organization. Therefore, no external dataset citation, repository DOI, or third-party data-source citation is applicable. De-identified data may be made available to qualified researchers for legitimate scientific purposes, subject to review and approval by the Institutional Review Board of Beirut Arab University. Requests should be submitted to the Institutional Review Board, Beirut Arab University, Beirut, Lebanon, at irb@bau.edu.lb. The Institutional Review Board will assess whether the proposed use is consistent with the original ethical approval, informed-consent conditions, and participant confidentiality protections.”

3- Your ethics statement should only appear in the Methods section of your manuscript. If your ethics statement is written in any section besides the Methods, please move it to the Methods section and delete it from any other section. Please ensure that your ethics statement is included in your manuscript, as the ethics statement entered into the online submission form will not be published alongside your manuscript.

Author’s response:

Thank you for this important comment. We confirm that the ethics statement has been retained within the Methods section under the subsection “Ethical considerations.” The ethics statement was reviewed to ensure that it includes the ethical approval source, approval date, informed-consent procedure, voluntary nature of participation, and confidentiality safeguards. Any duplicate ethics statement appearing elsewhere in the manuscript, including the end-matter declarations, has been removed.

4- Please include captions for your Supporting Information files at the end of your manuscript, and update any in-text citations to match accordingly.

Author’s response:

Thank you for this important comment. We have added a Supporting Information section at the end of the manuscript and included a caption for the supplementary appendix containing the full study questionnaire. The supporting file has been labelled as S1 Appendix, and the in-text citation in the Methods section has been revised accordingly. The revised citation and caption are as follows:

Questionnaire development and structure

“A self-administered questionnaire was developed by the research team following a review of relevant literature [5,6,8,35]. It consisted of 39 closed-ended questions covering sociodemographic characteristics, menstrual history, premenstrual symptoms, lifestyle and psychosocial factors, and PMS management practices. The questionnaire was administered in English, and the PSST, BSMAS, and PSS-4 were used in their original English versions. The full questionnaire is provided in S1 Appendix.”

Supporting information

“S1 Appendix. Study questionnaire. Self-administered questionnaire used to collect sociodemographic, menstrual, lifestyle, psychosocial, war-exposure, and premenstrual syndrome management-related data from female university students in Lebanon.”

5- If the reviewer comments include a recommendation to cite specific previously published works, please review and evaluate these publications to determine whether they are relevant and should be cited. There is no requirement to cite these works unless the editor has indicated otherwise.

Author’s response:

Thank you for this clarification. We carefully reviewed all references suggested by the reviewers and evaluated their relevance to the study background, methodology, and interpretation of findings. We included only those publications that were directly relevant to the revised manuscript. References that were not directly applicable to the scope or objectives of the present study were not added.

Additional Editor Comments:

1- Most sections of the manuscript need to be shortened.

Author’s response:

Thank you for this valuable comment. We carefully revised the manuscript to improve conciseness while preserving the essential methodological details, key findings, and scientific interpretation. The introduction was shortened by reducing detailed pathophysiological explanations and consolidating the overview of PMS management practices. The Methods section was condensed by simplifying the descriptions of validated assessment tools, questionnaire development, data collection, data analysis, and operational definitions. In the Results section, repetitive narrative descriptions of tabulated findings were reduced, and detailed information on management practices was consolidated to emphasize the most clinically relevant findings. The Discussion was also substantially shortened by reducing repetition, streamlining comparisons with previous studies, and focusing on the independent predictors and principal management findings. These revisions improved the overall clarity, flow, and readability of the manuscript.

2- Some tables could be merged together into one table. For example, the score of a certain scale can be used instead of showing each factor by itself (when applicable).

Author’s response:

Thank you for this valuable comment. We reviewed the tables to reduce unnecessary detail and improve the clarity of the main manuscript. Item-level results for the Bergen Social Media Addiction Scale and the Perceived Stress Scale-4 were moved to the Supporting Information, while their total scores were retained in the main text. In addition, detailed PMDD-specific regression analyses and the perceived effectiveness of lifestyle modifications were transferred to supplementary tables. These revisions reduced table length and repetition while preserving the key analyses and transparency of the study findings.

3- Better to mention the name of the university, as this would give a clearer understanding for readers.

Author’s response:

Thank you for this helpful comment. We agree that identifying the study setting provides clearer context for readers. Accordingly, the name of the university has been added to the Methods section under “Study design and setting” and is now specified as Beirut Arab University, Lebanon. As follows:

Study design and setting

“An observational cross-sectional study using convenience sampling was conducted among female students at Beirut Arab University, Lebanon, from April 20 to May 8, 2026. Beirut Arab University has four campuses distributed across Lebanon, and data were collected from students at all four campuses. The university enrolls students from diverse Lebanese regions and socioeconomic and cultural backgrounds.”

Reviewers’ comments:

Reviewer #1

1- The manuscript states that the study was conducted among female students at “a university in Lebanon” using convenience sampling, but the participating university is not clearly identified in the Methods section. Please specify the name of the university or clarify whether the data were collected from only one institution. If only one university was included, the authors should justify why other universities were not included and acknowledge that this limits the generalizability of the findings to all female university students in Lebanon.

Author’s response:

Thank you for this important comment. We have clarified in the Methods section that the study was conducted at Beirut Arab University, Lebanon. Although the study involved a single university, Beirut Arab University has four campuses distributed across Lebanon, and data were collected from female students at all four campuses. This approach enabled the inclusion of students from diverse geographical regions and socioeconomic and cultural backgrounds. Nevertheless, we acknowledge that the use of convenience sampling within one university may limit the generalizability of the findings to all female university students in Lebanon. This limitation has been added to the manuscript and the study design and setting has been adjusted as follows:

Study design and setting

“An observational cross-sectional study using convenience sampling was conducted among female students at Beirut Arab University, Lebanon, from April 20 to May 8, 2026. Beirut Arab University has four campuses distributed across Lebanon, and data were collected from students at all four campuses. The university enrolls students from diverse Lebanese regions and socioeconomic and cultural backgrounds.”

Study limitations

“The study was conducted among female students at a single university in Lebanon. Although data were collected across all four campuses of Beirut Arab University and included students from diverse geographical regions and socioeconomic backgrounds, the use of convenience sampling within one institution may limit the generalizability of the findings to all female university students in Lebanon.”

2- The inclusion criteria currently mention women of reproductive age, 18–45 years, but they do not explicitly state that participants had to be university students. Since the target population is female university students, this should be added to the inclusion criteria.

Author’s response:

Thank you for this important comment. We have revised the inclusion criteria to explicitly state that eligible participants were female university students aged 18–45 years who were enrolled at Beirut Arab University during the study period, as follows:

Inclusion and exclusion criteria

“The inclusion criteria were female students enrolled at Beirut Arab University, aged 18–45 years.”

3- The exclusion criteria include gynecological conditions, thyroid disorders, diagnosed mental health condition etc…. However, if they were self-reported, this should be acknowledged in the Limitations section as a source of recall bias and misclassification bias.

Author’s response:

Thank you for this important comment. The exclusion criteria, including gynecological conditions, thyroid disorders, diagnosed mental health conditions, pregnancy or breastfeeding, and hormonal contraceptive use, were based on self-reported information collected through the questionnaire. We have clarified this in the Methods section and added the potential for recall and misclassification bias related to self-reported exclusion criteria to the Limitations section, as follows:

Inclusion and exclusion criteria

“Participants were excluded if they reported gynecological conditions such as endometriosis, uterine fibroids, uterine polyps, polycystic ovary syndrome (PCOS), chronic pelvic inflammatory disease, or amenorrhea. Additionally, females reporting medical conditions, including thyroid disorders or diagnosed mental health conditions (e.g., anxiety or depression), as well as those currently using hormonal medications for contraception, were excluded. Pregnant or breastfeeding women were also excluded.”

Study limitations

“In addition, the cross-sectional design and reliance on retrospective self-reporting may have increased the possibility of recall bias. Self-reported medical and gynecological conditions used to determine eligibility may also have resulted in misclassification of some participants.”

4- The authors should state the language in which the questionnaire was distributed. If the questionnaire was administered in Arabic, is the Arabic validated versions of the PSST, BSMAS, and PSS-4 were used, and whether these versions were validated in Lebanon or in Arabic-speaking populations. If translation was performed by the research team, the translation/back-translation process should be described.

Author’s response:

Thank you for this important comment. We have clarified in the Methods section that the questionnaire was administered in English. Therefore, Arabic versions or translation/back-translation procedures were not applicable. The PSST, BSMAS, and PSS-4 were administered in their original English versions, as the study population comprised university students able to complete the questionnaire in English. The questionnaire development and structure section was adjusted as follows:

Questionnaire development and structure

“A self-administered questionnaire was developed by the research team following a review of relevant literature [5,6,8,35]. It consisted of 39 closed-ended questions covering sociodemograp

Attachment Submitted filename: 2-Responses to reviewers Comments PO RI.docx

# Peer review: Decision Letter 1

**DOI:** 10.1371/journal.pone.0354807.r003

**Authors:** Jad El Masri

29 Jun 2026

PONE-D-26-25846R1

Prevalence, risk factors, and management practices of premenstrual syndrome among female university students in Lebanon: an observational cross-sectional study

PLOS One

Dear Dr. Itani,

Thank you for submitting your manuscript to PLOS ONE. After careful consideration, we feel that it has merit but does not fully meet PLOS ONE’s publication criteria as it currently stands. Therefore, we invite you to submit a revised version of the manuscript that addresses the points raised during the review process.

**I congratulate the authors as the manuscript has clearly improved. Below are some minor comments, before the manuscript can move forward. Best of luck**

Please submit your revised manuscript by Jul 29 2026 11:59PM. If you will need more time than this to complete your revisions, please reply to this message or contact the journal office at plosone@plos.org. When you're ready to submit your revision, log on to https://www.editorialmanager.com/pone/ and select the 'Submissions Needing Revision' folder to locate your manuscript file.

Please include the following items when submitting your revised manuscript:

- A letter that responds to each point raised by the academic editor and reviewer(s). You should upload this letter as a separate file labeled 'Response to Reviewers'.
- A marked-up copy of your manuscript that highlights changes made to the original version. You should upload this as a separate file labeled 'Revised Manuscript with Track Changes'.
- An unmarked version of your revised paper without tracked changes. You should upload this as a separate file labeled 'Manuscript'.

If you would like to make changes to your financial disclosure, please include your updated statement in your cover letter. Guidelines for resubmitting your figure files are available below the reviewer comments at the end of this letter.

If applicable, we recommend that you deposit your laboratory protocols in protocols.io to enhance the reproducibility of your results. Protocols.io assigns your protocol its own identifier (DOI) so that it can be cited independently in the future. For instructions see: https://journals.plos.org/plosone/s/submission-guidelines#loc-laboratory-protocols. Additionally, PLOS ONE offers an option for publishing peer-reviewed Lab Protocol articles, which describe protocols hosted on protocols.io. Read more information on sharing protocols at https://plos.org/protocols?utm_medium=editorial-email&utm_source=authorletters&utm_campaign=protocols.

As the corresponding author, your ORCID iD is verified in the submission system and will appear in the published article. PLOS supports the use of ORCID, and we encourage all coauthors to register for an ORCID iD and use it as well. Please encourage your coauthors to verify their ORCID iD within the submission system before final acceptance, as unverified ORCID iDs will not appear in the published article. *Only*  the individual author can complete the verification step; PLOS staff *cannot*  verify ORCID iDs on behalf of authors.

We look forward to receiving your revised manuscript.

Kind regards,

Jad El Masri

Academic Editor

PLOS One

Journal Requirements:

If the reviewer comments include a recommendation to cite specific previously published works, please review and evaluate these publications to determine whether they are relevant and should be cited. There is no requirement to cite these works unless the editor has indicated otherwise.

Please review your reference list to ensure that it is complete and correct. If you have cited papers that have been retracted, please include the rationale for doing so in the manuscript text, or remove these references and replace them with relevant current references. Any changes to the reference list should be mentioned in the rebuttal letter that accompanies your revised manuscript. If you need to cite a retracted article, indicate the article’s retracted status in the References list and also include a citation and full reference for the retraction notice.

Additional Editor Comments (if provided):

I am pleasured to re-review this manuscript. I believe that the authors succeeded in making substantial improvements to their work. I still have some comments/suggestions that can help improve the work, before it can move forward.

Please find below my comments:

1- The introduction/discussion need to be shortened.

2- The limitations section could benefit from mentioning that BAU does not represent the Lebanese population, especially since it is a private university. Usually, the financial state is a major stress factor, so the chosen population may differ from other populations, such as public university students. Despite not being a major point, the paper would benefit from adding this explanation into the limitations.

3- Similar to figure 2, I suggest making similar representation for all the scales used. This is needed to give the readers general idea about the levels in the sample targeted, before delving into comparisons.

4- Tables showing frequency of answers on each item of the scale could be moved to supplementary, and those having univariate and multivariate could be in the main manuscript instead of the supplementary, as these give more information and offer greater value to readers.

Looking forward to reading the revised version

Best of luck

[Note: HTML markup is below. Please do not edit.]

Reviewers' comments:

Reviewer's Responses to Questions

**Comments to the Author**

1. If the authors have adequately addressed your comments raised in a previous round of review and you feel that this manuscript is now acceptable for publication, you may indicate that here to bypass the “Comments to the Author” section, enter your conflict of interest statement in the “Confidential to Editor” section, and submit your "Accept" recommendation.

Reviewer #2: All comments have been addressed

**********

2. Is the manuscript technically sound, and do the data support the conclusions?

The manuscript must describe a technically sound piece of scientific research with data that supports the conclusions. Experiments must have been conducted rigorously, with appropriate controls, replication, and sample sizes. The conclusions must be drawn appropriately based on the data presented.

Reviewer #2: Yes

**********

3. Has the statistical analysis been performed appropriately and rigorously?

Reviewer #2: Yes

**********

4. Have the authors made all data underlying the findings in their manuscript fully available?

The PLOS Data policy requires authors to make all data underlying the findings described in their manuscript fully available without restriction, with rare exception (please refer to the Data Availability Statement in the manuscript PDF file). The data should be provided as part of the manuscript or its supporting information, or deposited to a public repository. For example, in addition to summary statistics, the data points behind means, medians and variance measures should be available. If there are restrictions on publicly sharing data—e.g. participant privacy or use of data from a third party—those must be specified.

Reviewer #2: No

**********

5. Is the manuscript presented in an intelligible fashion and written in standard English?

PLOS ONE does not copyedit accepted manuscripts, so the language in submitted articles must be clear, correct, and unambiguous. Any typographical or grammatical errors should be corrected at revision, so please note any specific errors here.

Reviewer #2: Yes

**********

6. Review Comments to the Author

Please use the space provided to explain your answers to the questions above. You may also include additional comments for the author, including concerns about dual publication, research ethics, or publication ethics. (Please upload your review as an attachment if it exceeds 20,000 characters)

Reviewer #2: I congratulate authors on the progress made. I believe that making some minor changes would make the paper good to go.

1- The keywords could benefit from adding "University students" and removing abbreviated words as "SDG"

2- I agree that some tables should be removed to the supplementary part, but those having univariate and multivariate are worth keeping in my opinion. Those specifiying individual questions in the scale could be replaced with a figure showing the population's level, and these tables could be moved to the supplementary part.

3- The introduction could benefit from shortening, and the discussion needs to be shortened a lot (11 pages in its current form).

I believe that the manuscript is interesting and offers an addition to the literature after making these minor changes.

Good luck

**********

7. PLOS authors have the option to publish the peer review history of their article (what does this mean?). If published, this will include your full peer review and any attached files.

If you choose “no”, your identity will remain anonymous but your review may still be made public.

**Do you want your identity to be public for this peer review?**  For information about this choice, including consent withdrawal, please see our Privacy Policy.

Reviewer #2: No

**********

[NOTE: If reviewer comments were submitted as an attachment file, they will be attached to this email and accessible via the submission site. Please log into your account, locate the manuscript record, and check for the action link "View Attachments". If this link does not appear, there are no attachment files.]

To ensure your figures meet our technical requirements, please review our figure guidelines: https://journals.plos.org/plosone/s/figures

You may also use PLOS’s free figure tool, NAAS, to help you prepare publication quality figures: https://journals.plos.org/plosone/s/figures#loc-tools-for-figure-preparation.

NAAS will assess whether your figures meet our technical requirements by comparing each figure against our figure specifications.

# Author comment: Author response to Decision Letter 2

**DOI:** 10.1371/journal.pone.0354807.r004

1 Jul 2026

Dear Prof. Jad El Masri

Academic Editor

PLOS One

PONE-D-26-25846R1

Editor’s comments

I congratulate the authors as the manuscript has clearly improved. Below are some minor comments, before the manuscript can move forward. Best of luck

I am pleasured to re-review this manuscript. I believe that the authors succeeded in making substantial improvements to their work. I still have some comments/suggestions that can help improve the work, before it can move forward.

Thank you for giving us the opportunity to submit a second revised version of our manuscript entitled “Prevalence, risk factors, and management practices of premenstrual syndrome among female university students in Lebanon: an observational cross-sectional study” to PLOS One.

We sincerely appreciate the time and effort that you and the reviewers have dedicated to re-evaluating our manuscript. We are especially grateful for your positive assessment, acknowledging the substantial improvements made to our work. We highly value the constructive comments and suggestions provided, which have further strengthened the quality and clarity of the manuscript.

We have carefully considered all of the remaining comments and suggestions and have incorporated the recommended revisions throughout the manuscript. All modifications have been highlighted in the revised version for ease of review.

Please see below, in blue, for point-by-point responses to the comments and concerns.

Please find below my comments:

1. The introduction/discussion need to be shortened.

Authors’ response: Thank you for this constructive suggestion and for the positive evaluation of our manuscript. In response, we have carefully revised the manuscript to improve its conciseness and readability. Specifically, the Introduction has been streamlined by removing redundant background information and retaining only the most relevant literature to better establish the study rationale. In addition, the Discussion has been substantially shortened by eliminating repetitive statements, reducing unnecessary descriptive comparisons, and focusing on the interpretation and implications of the principal findings.

2. The limitations section could benefit from mentioning that BAU does not represent the Lebanese population, especially since it is a private university. Usually, the financial state is a major stress factor, so the chosen population may differ from other populations, such as public university students. Despite not being a major point, the paper would benefit from adding this explanation into the limitations.

Authors’ response: We sincerely thank you for this valuable suggestion. We agree that the study population may not fully represent all female university students in Lebanon, particularly those enrolled in public universities or institutions with different socioeconomic profiles. Accordingly, we have revised the limitations section to acknowledge that the study was conducted at a single private university, which may limit the generalizability of the findings. We also noted that socioeconomic differences, including financial stressors, may influence the prevalence and experience of premenstrual syndrome and should be considered when interpreting the results. We have further recommended that future studies include students from both public and private universities across different regions of Lebanon to enhance the representativeness and generalizability of the findings.

Revised text in the manuscript, under the study limitations section:

“The study was conducted among female students at a single university in Lebanon. Although data were collected across all four campuses of Beirut Arab University and included students from diverse geographical regions, the use of convenience sampling within one institution may limit the generalizability of the findings to all female university students in Lebanon. Furthermore, because Beirut Arab University is a private institution, the study population may not fully represent students enrolled in public universities or other higher education institutions in Lebanon. Differences in socioeconomic characteristics and financial stressors between students attending public and private universities may influence the prevalence and severity of PMS and PMDD and should therefore be considered when interpreting the findings.”

“Future multicenter studies involving students from diverse educational institutions, including both public and private universities, and different geographic regions across Lebanon are warranted to provide more representative national estimates and enhance the generalizability of the findings.”

3. Similar to figure 2, I suggest making similar representation for all the scales used. This is needed to give the readers general idea about the levels in the sample targeted, before delving into comparisons.

Authors’ Response: We sincerely thank you for this valuable suggestion. In response to your and reviewer # 2 recommendation, we have added graphical representations of the distributions of participants' responses for both the Bergen Social Media Addiction Scale (BSMAS) and the Perceived Stress Scale-4 (PSS-4) by including Figures 3 and 4, respectively. These figures provide readers with a clear overview of the distribution of responses across the study sample before presenting the comparative and regression analyses. The corresponding descriptions and citations have also been incorporated into the Results section.

4. Tables showing frequency of answers on each item of the scale could be moved to supplementary, and those having univariate and multivariate could be in the main manuscript instead of the supplementary, as these give more information and offer greater value to readers.

Looking forward to reading the revised version

Best of luck

Authors’ response: We sincerely thank you for this valuable suggestion. In response, we have reorganized the presentation of the tables to improve the readability and overall flow of the manuscript. Specifically, the detailed tables presenting the frequency distributions of responses to the individual questionnaire items (PSST; originally Tables 3 and 4) have been moved to the Supplementary Material, while the univariate and multivariable regression analysis tables related to the predictors of PMDD have been moved to the main manuscript, as these represent the primary analytical findings of the study and provide greater value to readers. Accordingly, the table numbering and all corresponding in-text citations have been revised throughout the manuscript to reflect these changes. We appreciate this helpful recommendation, which has enhanced the clarity and organization of the manuscript.

[Note: HTML markup is below. Please do not edit.]

Reviewers' comments:

Reviewer's Responses to Questions

Comments to the Author

1. If the authors have adequately addressed your comments raised in a previous round of review and you feel that this manuscript is now acceptable for publication, you may indicate that here to bypass the “Comments to the Author” section, enter your conflict of interest statement in the “Confidential to Editor” section, and submit your "Accept" recommendation.

Reviewer #2: All comments have been addressed

________________________________________

2. Is the manuscript technically sound, and do the data support the conclusions?

The manuscript must describe a technically sound piece of scientific research with data that supports the conclusions. Experiments must have been conducted rigorously, with appropriate controls, replication, and sample sizes. The conclusions must be drawn appropriately based on the data presented.

Reviewer #2: Yes

________________________________________

3. Has the statistical analysis been performed appropriately and rigorously?

Reviewer #2: Yes

________________________________________

4. Have the authors made all data underlying the findings in their manuscript fully available?

The PLOS Data policy requires authors to make all data underlying the findings described in their manuscript fully available without restriction, with rare exception (please refer to the Data Availability Statement in the manuscript PDF file). The data should be provided as part of the manuscript or its supporting information, or deposited to a public repository. For example, in addition to summary statistics, the data points behind means, medians and variance measures should be available. If there are restrictions on publicly sharing data—e.g. participant privacy or use of data from a third party—those must be specified.

Reviewer #2: No

Authors' Response: We sincerely thank you for highlighting this important point. In response, we have now made the underlying data supporting the findings of this study fully available by providing the complete dataset as Supporting Information in accordance with the PLOS ONE Data Availability Policy. The Data Availability Statement has also been revised accordingly to reflect this update.

As follow: “Availability of data and materials: All data underlying the findings of this study are fully available without restriction. The complete de-identified dataset has been provided as S1 Dataset in the Supporting Information.”

________________________________________

5. Is the manuscript presented in an intelligible fashion and written in standard English?

PLOS ONE does not copyedit accepted manuscripts, so the language in submitted articles must be clear, correct, and unambiguous. Any typographical or grammatical errors should be corrected at revision, so please note any specific errors here.

Reviewer #2: Yes

________________________________________

6. Review Comments to the Author

Please use the space provided to explain your answers to the questions above. You may also include additional comments for the author, including concerns about dual publication, research ethics, or publication ethics. (Please upload your review as an attachment if it exceeds 20,000 characters)

Reviewer #2: I congratulate authors on the progress made. I believe that making some minor changes would make the paper good to go.

Authors' Response: We sincerely thank you for the careful re-evaluation of our manuscript and for the encouraging and positive feedback. We greatly appreciate the recognition of the progress made in improving the manuscript. We have carefully considered all of the remaining comments and suggestions and have revised the manuscript accordingly. Our detailed point-by-point responses are provided below.

1. The keywords could benefit from adding "University students" and removing abbreviated words as "SDG"

Authors' Response: We sincerely thank you for this helpful suggestion. In response, we have revised the keywords by removing the abbreviation “SDG” and by adding “University Students”.

2. I agree that some tables should be removed to the supplementary part, but those having univariate and multivariate are worth keeping in my opinion. Those specifiying individual questions in the scale could be replaced with a figure showing the population's level, and these tables could be moved to the supplementary part.

Authors' Response: Thank you for this valuable suggestion. In response, we have reorganized the presentation of the results to improve the readability and overall flow of the manuscript. Specifically, the detailed tables presenting the frequency distributions of responses to the individual PSST items (formerly Tables 3 and 4) have been moved to the Supplementary Material. In addition, we have added Figures 3 and 4, which graphically present the distributions of participants' responses to the Bergen Social Media Addiction Scale (BSMAS) and the Perceived Stress Scale-4 (PSS-4), respectively, providing readers with an overall overview of the response distributions before the comparative analyses. Furthermore, the univariate and multivariable regression analysis tables have been retained in the main manuscript, as recommended. The table numbering and corresponding in-text citations have also been updated throughout the manuscript to reflect these changes.

3. The introduction could benefit from shortening, and the discussion needs to be shortened a lot (11 pages in its current form).

I believe that the manuscript is interesting and offers an addition to the literature after making these minor changes.

Good luck

Authors' Response: Thank you for this constructive suggestion and for the positive evaluation of our manuscript. In response, we have carefully revised the manuscript to improve its conciseness and readability. Specifically, the Introduction has been streamlined by removing redundant background information and retaining only the most relevant literature to better establish the study rationale. In addition, the Discussion has been substantially shortened by eliminating repetitive statements, reducing unnecessary descriptive comparisons, and focusing on the interpretation and implications of the principal findings.

________________________________________

7. PLOS authors have the option to publish the peer review history of their article (what does this mean?). If published, this will include your full peer review and any attached files.

If you choose “no”, your identity will remain anonymous but your review may still be made public.

Do you want your identity to be public for this peer review? For information about this choice, including consent withdrawal, please see our Privacy Policy.

Reviewer #2: No

________________________________________

Attachment Submitted filename: Responses to Reviewers Comments - Second Round PO.docx

# Editor report: Decision Letter 2

**DOI:** 10.1371/journal.pone.0354807.r005

**Authors:** Jad El Masri

14 Jul 2026

Prevalence, risk factors, and management practices of premenstrual syndrome among female university students in Lebanon: an observational cross-sectional study

PONE-D-26-25846R2

Dear Dr. Ritani

We’re pleased to inform you that your manuscript has been judged scientifically suitable for publication and will be formally accepted for publication once it meets all outstanding technical requirements.

Within one week, you’ll receive an e-mail detailing the required amendments. When these have been addressed, you’ll receive a formal acceptance letter and your manuscript will be scheduled for publication.

An invoice will be generated when your article is formally accepted. Please note, if your institution has a publishing partnership with PLOS and your article meets the relevant criteria, all or part of your publication costs will be covered. Please make sure your user information is up-to-date by logging into Editorial Manager at Editorial Manager® and clicking the ‘Update My Information' link at the top of the page. For questions related to billing, please contact billing support.

If your institution or institutions have a press office, please notify them about your upcoming paper to help maximize its impact. If they’ll be preparing press materials, please inform our press team as soon as possible -- no later than 48 hours after receiving the formal acceptance. Your manuscript will remain under strict press embargo until 2 pm Eastern Time on the date of publication. For more information, please contact onepress@plos.org.

Kind regards,

Jad El Masri

Academic Editor

PLOS One

Additional Editor Comments (optional):

Reviewers' comments:

# Editor report: Acceptance letter

**DOI:** 10.1371/journal.pone.0354807.r006

**Authors:** Jad El Masri

PONE-D-26-25846R2

PLOS One

Dear Dr. Itani,

I'm pleased to inform you that your manuscript has been deemed suitable for publication in PLOS One. Congratulations! Your manuscript is now being handed over to our production team.

At this stage, our production department will prepare your paper for publication. This includes ensuring the following:

* All references, tables, and figures are properly cited

* All relevant supporting information is included in the manuscript submission,

* There are no issues that prevent the paper from being properly typeset

You will receive further instructions from the production team, including instructions on how to review your proof when it is ready. Please keep in mind that we are working through a large volume of accepted articles, so please give us a few days to review your paper and let you know the next and final steps.

Lastly, if your institution or institutions have a press office, please let them know about your upcoming paper now to help maximize its impact. If they'll be preparing press materials, please inform our press team within the next 48 hours. Your manuscript will remain under strict press embargo until 2 pm Eastern Time on the date of publication. For more information, please contact onepress@plos.org.

You will receive an invoice from PLOS for your publication fee after your manuscript has reached the completed accept phase. If you receive an email requesting payment before acceptance or for any other service, this may be a phishing scheme. Learn how to identify phishing emails and protect your accounts at https://explore.plos.org/phishing.

If we can help with anything else, please email us at customercare@plos.org.

Thank you for submitting your work to PLOS ONE and supporting open access.

Kind regards,

PLOS ONE Editorial Office Staff

on behalf of

Dr. Jad El Masri

Academic Editor

PLOS One
